# Adapting hippocampus multi-scale place field distributions in cluttered environments optimizes spatial navigation and learning

**DOI:** 10.3389/fncom.2022.1039822

**Published:** 2022-12-12

**Authors:** Pablo Scleidorovich, Jean-Marc Fellous, Alfredo Weitzenfeld

**Affiliations:** ^1^Department of Computer Science and Engineering, University of South Florida, Tampa, FL, United States; ^2^Department of Psychology and Biomedical Engineering, University of Arizona, Tucson, AZ, United States

**Keywords:** hippocampus, spatial navigation, multi-scale, place cells, spatial learning, spatial cognition, reinforcement learning

## Abstract

Extensive studies in rodents show that place cells in the hippocampus have firing patterns that are highly correlated with the animal's location in the environment and are organized in layers of increasing field sizes or scales along its dorsoventral axis. In this study, we use a spatial cognition model to show that different field sizes could be exploited to adapt the place cell representation to different environments according to their size and complexity. Specifically, we provide an in-depth analysis of how to distribute place cell fields according to the obstacles in cluttered environments to optimize learning time and path optimality during goal-oriented spatial navigation tasks. The analysis uses a reinforcement learning (RL) model that assumes that place cells allow encoding the state. While previous studies have suggested exploiting different field sizes to represent areas requiring different spatial resolutions, our work analyzes specific distributions that adapt the representation to the environment, activating larger fields in open areas and smaller fields near goals and subgoals (e.g., obstacle corners). In addition to assessing how the multi-scale representation may be exploited in spatial navigation tasks, our analysis and results suggest place cell representations that can impact the robotics field by reducing the total number of cells for path planning without compromising the quality of the paths learned.

## 1. Introduction

The study of spatial cognition requires understanding how space is represented in the brain and how these representations are formed, used, and maintained. Although early behavioral studies suggested the existence of a “cognitive map” in the brain (Tolman, 1948), it was not until 1971 that any light was shed regarding possible neural implementations.

Since 1971, electrophysiological studies have reported multiple types of neurons that encode spatial information in the brain, providing possible substrates for implementing the cognitive map. Initial studies reported the existence of “place cells” from recordings of individual pyramidal cells in the hippocampal substructures CA1 and CA3 (O'Keefe and Dostrovsky, 1971). Place cells are neurons whose activation is highly associated with the animal's position in space, forming compact firing fields dependent on local and distal cues but independent of the animal's bearings (O'Keefe and Nadel, 1978; McNaughton et al., 1996). Later on, Ranck discovered the existence of head direction cells that encoded allocentric orientation in the azimuthal plane resembling an internal compass (Ranck, 1984; Taube et al., 1990; Chen et al., 1994; Taube, 1998; Guzowski et al., 2004). As with place cells, the activity of head direction cells is driven both by visual cues and egocentric motion signals, the latter enabling orientation even when moving in darkness (Cho and Sharp, 2001; Rolls, 2005). More recently, Moser and Moser identified the existence of grid cells located in the entorhinal cortex as part of a “neural odometry” system for rat navigation (Fyhn et al., 2004; Hafting et al., 2005; Moser et al., 2008). Additionally, neurons have also been reported to encode environmental boundaries (border cells and boundary vector cells) (Savelli et al., 2008; Solstad et al., 2008; Lever et al., 2009), objects (object cells, object-trace cells, and obstacle-vector cells) (Deshmukh and Knierim, 2011; Deshmukh et al., 2012; Tsao et al., 2013; Hoydal et al., 2018; Andersson et al., 2021), and target goals and landmarks in the environment (Deshmukh and Knierim, 2013; Sarel et al., 2017).

Previous research shows that place cells have a multi-scale organization along the dorsoventral axis of the hippocampus, with dorsal place cells having smaller compact fields and ventral cells having larger, less stable fields (Jung and Wiener, 1994; Maurer et al., 2005; Kjelstrup et al., 2008; Keinath et al., 2014; Long et al., 2015). Initially, this difference was explained by assigning different roles to each region, with dorsal cells associated with spatial navigation and memory and ventral cells with planning, learning, and emotion (Fanselow and Dong, 2010; Poppenk et al., 2013; Strange et al., 2014). In contrast, newer studies suggest that ventral place cells are also involved in spatial navigation (de Hoz et al., 2003; Harland et al., 2017; Contreras et al., 2018).

In previous work, we developed a multi-scale spatial cognition model based on the differences between the dorsal and ventral hippocampus and the basal ganglia (Scleidorovich et al., 2020). The model implemented a reinforcement learning algorithm that learned a goal-oriented spatial navigation task based on theories suggesting that dopamine implements a reinforcement learning signal and that place cells may provide a basis set for computing value functions (Montague et al., 1996; Suri, 2002; Gustafson and Daw, 2011; Sutton and Barto, 2018). Experiments with the model assessed the benefits of using different scales for navigating open-field mazes with up to two obstacles by distributing place cell fields uniformly over space.

In this article, we update the multi-scale spatial cognition model and use it to study its behavior in complex, obstacle-rich environments. Particularly, we assess how the number of obstacles affects the learning for different fields sizes, we introduce metrics for evaluating the “relevance” of each scale for encoding value functions in multi-scale models, and we assess how to adapt the place cell field representation to the environment to enable more robust and efficient navigation. We hypothesize that areas near navigation goals and subgoals (i.e., obstacle corners) require high resolution and benefit from using an increased number of smaller fields to represent space. On the other hand, we hypothesize that areas further away require less resolution and benefit from using fewer, larger fields that can generalize experience quickly. As a result, we hypothesize that distributing place cells according to the environment can reduce the total number of cells used and the time required to learn a navigation task without decreasing navigation efficiency (i.e., without increasing the number of actions required to reach the goal).

This article presents the updated spatial navigation model and analyzes results from different experiments varying the place cell spatial distribution methods. The experiments were designed to investigate and assess the impact of place cell distributions on navigation and learning depending on environment configuration. Specifically, the experiments were designed to study: 1) the relationship between the number and size of uniform single-scale place fields and the number and configuration of obstacles in the environment, 2) the contribution of different scales in uniform multi-scale distributions based on the number and configuration of obstacles in the environment, 3) the impact of smaller place fields around goals and subgoals in the environment, and 4) the distribution of non-uniform multi-scale place cell fields to optimize all metrics simultaneously (number of cells, learning time, and navigation efficiency).

The main contributions of this article are:

1. A study for distributing multi-scale place cell fields for optimizing spatial navigation founded on empirical and theoretical computational background.
2. An analysis of how different field sizes interact with obstacles.
3. A proposal of how hierarchical reinforcement learning algorithms could leverage the proposed spatial representation.

This work suggests a possible methodology for distributing place cell fields in specific environments in order to exploit their multi-scale nature in reinforcement learning algorithms. This research is based on experimental studies in rats and computational models developed by our group, impacting both our understanding on place cell activiations and spatial navigation and learning in other domains, including autonomous robot systems.

In the rest of this article, Section 2 presents related work, Section 3 presents the research methods, Section 4 presents the experimental results, and Section 5 presents the discussion.

## 2. Related work

This section reviews related works in spatial cognition modeling. Our work assesses different place cell distribution methods for navigating cluttered environments using a reinforcement learning spatial cognition model based on the hippocampus. Due to the diverse topics, we divide the related works into three categories according to their aim, including using models to explain how the multi-scale place cell representation is formed, developing multi-scale models for navigation, and developing models for navigating complex and cluttered environments. In addition the following subsections, we note that related work by Tessereau et al. (2021) provides a survey on spatial cognition models inspired by the hippocampus (HC), while Madl et al. (2015) reviews cognitive models of spatial memory, categorizing them according to the environment's complexity and the possibility of mapping them to neural substrates.

### 2.1. Field size explanatory models

In general, multi-scale computational models have been developed to explain the differences between dorsal and ventral HC. In Neher et al. (2017), the authors argue that realistic place field sizes cannot be explained by feedforward models using grid cells as the only input. Instead, the authors propose adding nonspatial information and using recurrent connections between place cells to account for realistic field sizes. Similarly, Lyttle et al. (2013) extend the work by de Almeida et al. (2009) to assess whether nonspatial inputs can explain the field size differences observed between dorsal and ventral HC. As a result, their model suggests a shift in the type of information encoded by each region. In Navratilova et al. (2012), a model of grid cells is described based on attractor dynamics. The model can account for phase precession and the difference between grid cell field sizes in the medial entorhinal cortex (MEC). Although this is not a model of the hippocampus, the multi-scale representation in HC is believed to depend on the MEC's multi-scale representation. In Burgess et al. (2000) and Barry et al. (2006), the authors present a computational model of place cells that use boundary vector cells as input. The model can explain how place cells react to some environmental manipulations such as environment rescalings or obstacle additions and removals. The model attributes larger field sizes to greater uncertainties when coding long distances to boundaries.

### 2.2. Multi-scale navigation models

In another group of articles, bioinspired multi-scale models have been developed to improve different navigation aspects. In Chen et al. (2013, 2014, 2015), Fan et al. (2017), and Hausler et al. (2020), authors developed a multi-scale model for localization based on the medial entorhinal cortex (MEC), using visual input to drive layers of grid cell-like objects. The model was compared against state-of-the-art localization algorithms from traditional robotics showing it could outperform them by recognizing more locations without losing precision. Additionally, the model was used to provide insights into the number of place field scales and sizes the brain should use. In Erdem and Hasselmo (2012, 2014), the authors describe a spatial cognition model mimicking preplay during sharp-wave ripples (Ólafsdóttir et al., 2018). The model was based on the MEC and HC and used multi-scale place cells to extend the distance covered by preplay sequences, thus allowing the model to plan paths farther away from goals. In Chalmers et al. (2016), the authors describe a multi-scale spatial cognition model inspired by the hippocampus combining model-based reinforcement learning, preplay-like processes, and context-driven remapping of place cells. Experiments with the model illustrate how the multi-scale representation allowed faster learning by reducing the computational requirements for adapting the agent to new or changing environments. In Llofriu et al. (2015) and Scleidorovich et al. (2020), the authors describe reinforcement learning multi-scale models for spatial cognition based on the difference between the dorsal and ventral hippocampus. The models use uniform distributions of place fields to assess the benefits of a multi-scale architecture regarding learning time, path optimality, and the number of cells. Experiments were performed in open mazes with few or no obstacles.

Although our article assesses methods to improve navigation using a multi-scale place field model, unlike the previous related works, this paper analyzes the effect of obstacles on place field distributions. In particular, we analyze how place fields should be distributed to support navigation in complex and obstacle-rich environments.

### 2.3. Navigation models in complex environments

Other articles assess how the brain may support navigation in complex and cluttered environments. In these studies, articles follow two main (complementary) approaches. One approach implements neurons that encode obstacle information, as observed in electrophysiological experiments (Savelli et al., 2008; Solstad et al., 2008; Lever et al., 2009; Deshmukh and Knierim, 2011; Deshmukh et al., 2012; Tsao et al., 2013; Hoydal et al., 2018; Andersson et al., 2021), while the other implements hierarchical reinforcement learning methods that add subgoals to tasks (Parr and Russell, 1997; Sutton et al., 1999; Dietterich, 2000; Barto and Mahadevan, 2003). In Llofriu et al. (2019), the authors use a multi-scale spatial cognition model in semi-dynamic environments. The model incorporates “object-interactive” place fields that enable learning different policies when obstacles are present by activating or deactivating fields when introducing intersecting obstacles. The model was used to reproduce rat experiments where inactivating dorsal or ventral hippocampus impaired open-field navigation only in cluttered environments. In Edvardsen et al. (2020), the authors describe a spatial cognition model capable of navigating toward goals in cluttered environments, exploiting unexplored novel shortcuts. The model implements grid cells to support vector navigation, border cells to allow obstacle avoidance, and place cells to use as a topological map along a preplay model to set subgoals when the agent gets stuck during vector navigation. In Botvinick et al. (2009), Botvinick (2012), and Botvinick and Weinstein (2014), the authors analyze hierarchical reinforcement learning methods and assess neural mechanisms that might allow their implementation in the brain by reviewing empirical findings. Similarly, Brunec and Momennejad (2022) analyze human fMRI recordings to assess whether the hippocampus and the prefrontal cortex may encode multi-scale predictive representations, as suggested by computational models using reinforcement learning's successor representation. In Chalmers et al. (2016), place cell preplay-like events are used to choose subgoals in a hierarchical reinforcement learning model. The resulting algorithm was used in semi-dynamic environments and allowed reducing learning times by generalizing knowledge across environments.

In our work, instead of neurons encoding obstacle information or hierarchical learning, we associate obstacle corners with subgoals and consider the benefits of adapting the number of place fields, their position, and their size according to the distance to the closest subgoal. This place field distribution method may complement hierarchical reinforcement learning models by providing a space representation that encodes subgoals naturally.

## 3. Research methods

To assess our hypotheses, we performed multiple experiments in simulated environments where a robot had to do the same goal-oriented task, using different place field distributions and obstacle configurations. The details are provided in the following sections.

### 3.1. Task

The task consisted in having an agent (simulated rat) learn to navigate a maze toward a single goal from multiple predefined starting locations. Note that both the goal and the set of starting locations varied according to the maze (see Section 3.2 for details).

**Figure 1:**
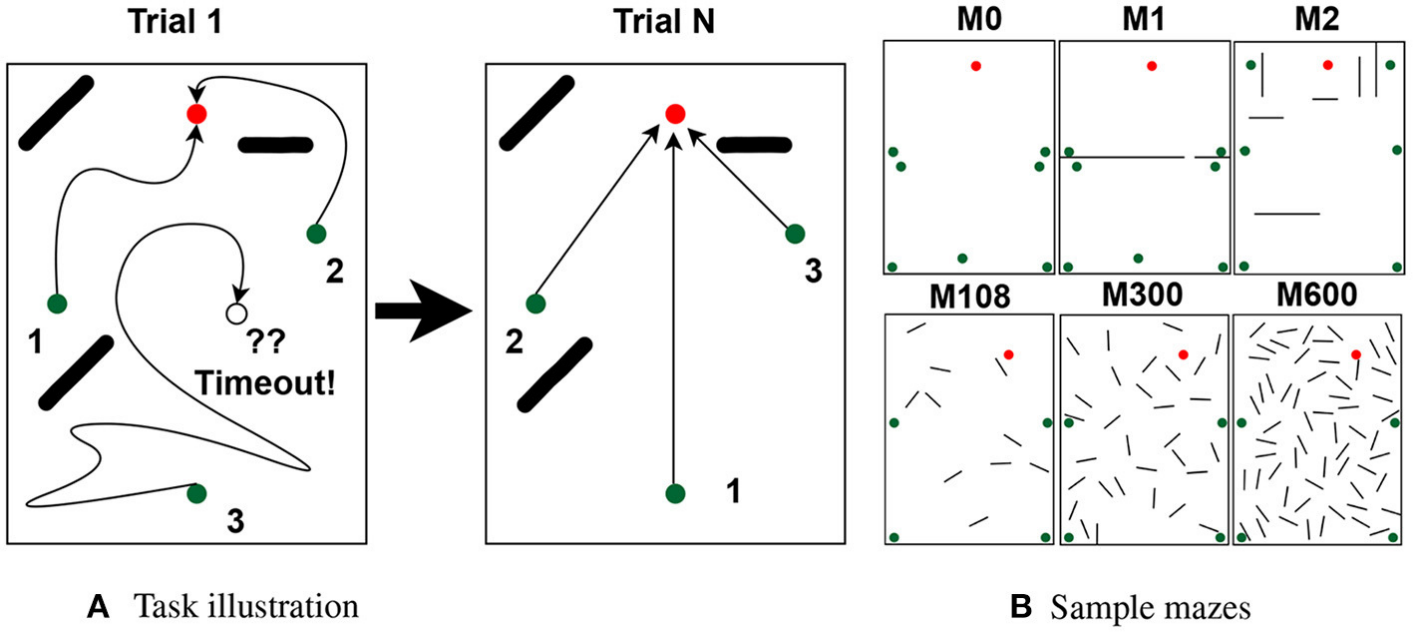
Task and mazes. **(A)** Rats have *N* trials to learn the path to the goal (red dots) from multiple starting locations (green dots). On each trial, the agent traverses the maze once per starting location in random order (path numbers). Traversals end by reaching the goal or a timeout. **(B)** Sample mazes used in the experiments.

Agents were given *N* trials to learn the shortest paths, where each trial corresponds to navigating the maze once from each starting location. The order of starting locations varied every trial and was chosen by sampling a random permutation from a uniform distribution. Each navigation began after placing the agent at the respective starting location and ended by reaching either the goal or a timeout. Rewards were given only at the goal, and timeouts were defined as performing 4,000 actions without reaching the goal. Note that the shortest paths measured 23 steps on average, leaving ample room for the agent to find the goal. The agents were considered to reach the goal when arriving at any position within 8 cm from the goal (the body of a rat is about 20 cm long). Figure 1A illustrates the task.

### 3.2. Mazes

In total, we used 63 mazes of identical dimensions (2.2 m by 3 m), each with its own goal, starting locations, and obstacles. Of the 63 mazes, 60 were generated automatically and were used to assess the effect of obstacles over different place field sizes. Automatically generated mazes had either 10, 20, ..., or 60 25 cm long obstacles (10 mazes per obstacle number). The other 3 mazes were generated manually to assess non-uniform distributions. Figure 1B shows the 3 handmade mazes (top row) and 3 of the 60 automatically generated mazes. See Supplementary Section 1 for a full description of the mazes and a discussion of how adding starting locations increases task difficulty.

### 3.3. Spatial navigation model

The paper describes a modified multi-scale spatial cognition model based on Scleidorovich et al. (2020). The following sections describe the model, highlighting the key differences between the original and latest model. Note that, throughout the document, indices i, j, t, and T represent place field i, action j, time t, and trial T, respectively.

#### 3.3.1. Overview

**Figure 2:**
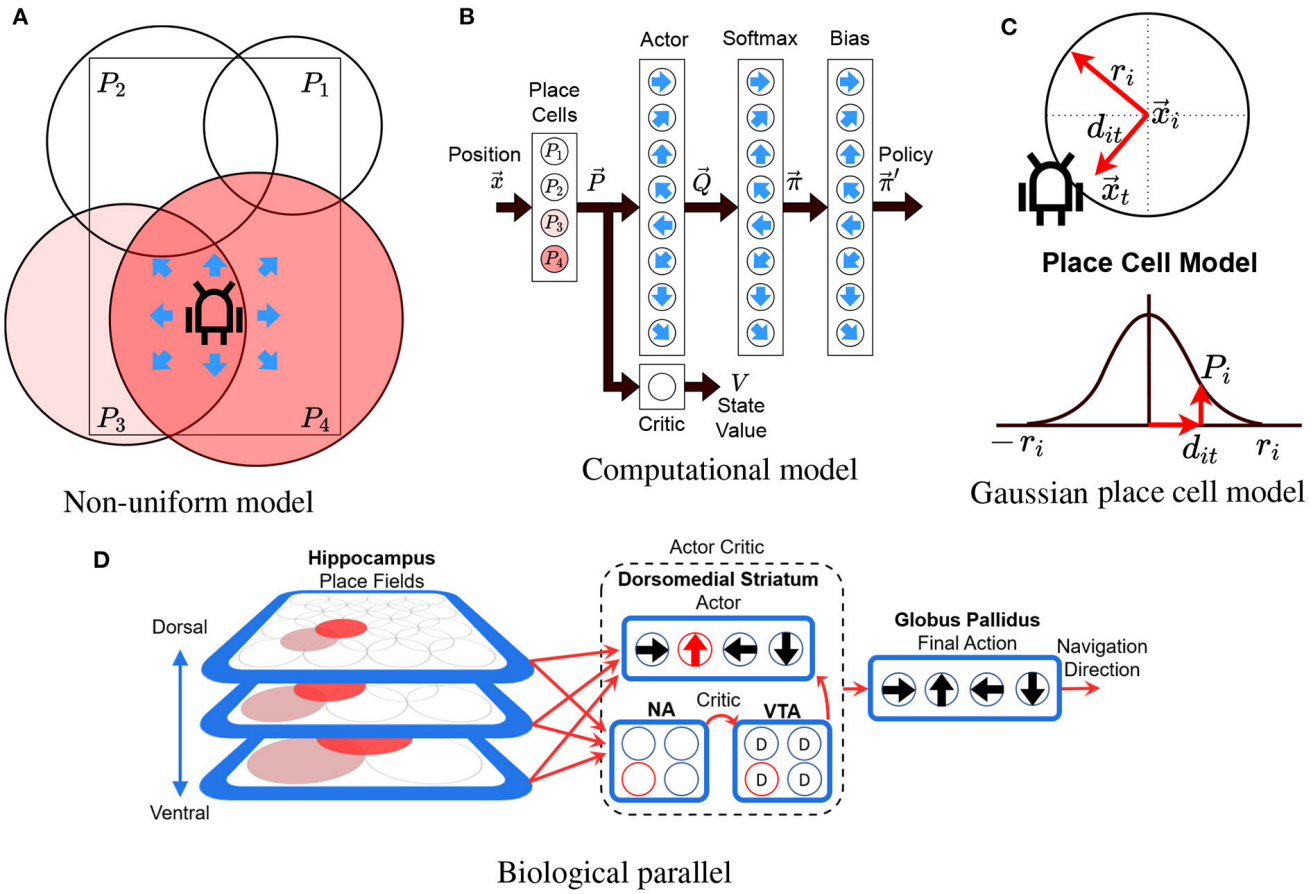
Model illustration. **(A)** The robot in a rectangular maze with 4 differently sized place cells (*P*_1_ to *P*_4_). Color illustrates the activity of each place cell. Arrows illustrate the 8 possible directions that the robot can take (actions). **(B)** The actor-critic network. Circles illustrate neurons and black arrows illustrate information flow between layers. Blue arrows indicate the action associated to each cell. Place cells are fully connected to the actor critic layers. The softmax and bias layers convert the actor's output into a set of probabilities for each action. **(C)** Variables involved in computing the gaussian place cell model (see Section 3.3.2). **(D)** Our model's biological counterpart. Red arrows show information flow while cells highlighted in red indicate active cells. D, NA, and VTA stand for dopamine, nucleus accumbens, and ventral tegmental area. This diagram is an adaption of our previous model described in "A Computational Model for a Multi-Goal Spatial Navigation Task inspired by Rodent Studies" by Llofriu et al., IJCNN, 2019, pp. 1–8.

The model uses an Actor-Critic RL algorithm with linear function approximation, using Gaussians as the radial basis functions and eligibility traces (Konda and Tsitsiklis, 1999; Sutton and Barto, 2018). The model's objective is to allow an agent (real or simulated robot) to learn to reach a goal from multiple starting locations on a maze. At each time step, the model uses the position of the robot ${\vec{x}}_{t}\in {\mathbb{R}}^{2}$ as input and chooses to perform one of eight possible allocentric actions *a*_*t*_ ∈ {0, ..., 7} as output. Action *j* represents moving one step (8 cm) in the cardinal direction $\theta_{j}=\frac{\pi }{4}j$. The computational model is illustrated in Figures 2A–C, and its pseudocode is shown in Supplementary Algorithm 1. Figure 2A illustrates the non-uniform place cell representation along with the robot and the possible actions, Figure 2B illustrates an overview of the actor-critic model (described in the following subsections), and Figure 2C illustrates the place cell model (described in Section 3.3.2).

The biological counterpart of our model's architecture is presented in Figure 2D. The model assumes that the basal ganglia enable the brain to perform reinforcement learning-like processes, using dopamine as a reinforcement signal (Montague et al., 1996; Suri, 2002; Sutton and Barto, 2018). Additionally, we assume that place cells encode the reinforcement learning state providing a basis for computing value functions (Gustafson and Daw, 2011). Using these hypotheses, the model provides the information from the hippocampus (HC) as input to a learning module comprised of the dopaminergic neurons of the ventral tegmental area (VTA), the dorsomedial striatum, and the ventral striatum (nucleus accumbens - NA). In particular, the different hippocampus place cells project their output to a value estimating network, with input relayed to the nucleus accumbens, VTA, and action selection structures in the dorsomedial striatum. Dopaminergic error signals are projected to the dorsomedial striatum, where they are used to learn the associations between situations (stimulus) and actions (response). All action selection information converges on a common structure for final action selection (Globus Pallidus), corresponding to navigation direction.

#### 3.3.2. Place cells

Our model represents place fields with normalized Gaussians that serve as the basis for the linear function approximators in the RL algorithm (Bugmann, 1998; Sutton and Barto, 2018). To compute the activity of a place cell, each place cell is assigned a circular field with center ${\vec{x}}_{i}$ and radius *r*_*i*_. Outside the radius, the activity is set to 0. Inside the radius, the activity is calculated by using the Gaussian kernel and then normalizing the results by the sum of all cells, as described in Equations (1) and (2). The place cell model is illustrated in Figure 2C.


(1) $$\begin{array}{l} {P^{\prime }}_{it}=\;\{\begin{array}{l} 0\;d_{it}<r_{i}\\ e^{-\frac{d_{it}^{2}}{r_{i}^{2}}ln(\alpha )}\text{ otherwise} \end{array}\; \end{array}$$

(2) $$\begin{array}{l} P_{it}=\frac{{P^{\prime }}_{it}}{\sum_{i}{P^{\prime }}_{it}}\; \end{array}$$

Where

- $P_{it}^{\prime }$ and *P_it_* represent the activity of place cell i at time t before and after normalization.
- $d_{it}=||{\vec{x}}_{t}-{\vec{x}}_{i}||$ is the Euclidean distance from ${\vec{x}}_{i}$ (the center of place field *i*) to ${\vec{x}}_{t}$ (the position of the agent at time *t*).
- *r_i_* is the radius of place cell *i*.
- α is a constant (set to 0.001) that represents the value of the Gaussian when *d_it_* = *r_i_*.
- *e* and *ln* are the exponential and natural logarithm functions, respectively.

#### 3.3.3. Function approximation

As we use a continuous state space ${\vec{x}}_{t}\in {\mathbb{R}}^{2}$, our model uses linear function approximators for both the actor and the critic to generalize the information gathered from discrete observations (Sutton and Barto, 2018). The approximators associate each place cell *i* with a set of parameters *V*_*i*_ and *Q*_*ij*_. Although not precisely the same, these parameters can be, respectively, thought of as representing the value at state ${\vec{x}}_{i}$ (i.e., the expectancy of future reward if starting from ${\vec{x}}_{i}$) and the actor's preference for performing action *a*_*j*_ at state ${\vec{x}}_{i}$. Using the place cells as kernels and the parameters, we compute the current state value and the preference for each action according to Equations (3) and (4).


(3) $$\begin{array}{l} V_{t}=\sum_{i}P_{it}V_{it} \end{array}$$

(4) $$\begin{array}{l} Q_{jt}=\sum_{i}P_{it}Q_{ijt} \end{array}$$

Where

- *V_t_* is the state value at time *t*.
- *V_it_* is the state value associated with place cell *i* at time *t*.
- *Q_jt_* is the preference for action *j* at time *t*.
- *Q_ijt_* is the preference for action *j* associated with place cell *i* at time *t*.

#### 3.3.4. Action selection

After computing the preference for each action, action selection is performed by converting the preferences into a set of probabilities according to Equations (5) and (6) and then sampling a random action from the resulting distribution.

Equation (5) computes an initial probability set π_*jt*_ from the action preferences by applying the softmax function but setting to 0 the probability of actions impeded by obstacles (see example in Supplementary Figure S2). Although we could allow the model to choose impeded actions, removing them prevents the robot from performing unnecessary actions and hitting obstacles.


(5) $$\begin{array}{l} \pi_{jt}=\;\frac{b_{jt}e^{Q_{jt}}\;}{\sum_{k}b_{kt}e^{Qkt}} \end{array}$$

Where

- π*_jt_* is the probability of performing action *j* at time *t* according to the actor's policy.
- *b_jt_* is a Boolean variable indicating whether action *j* can be performed at time *t* or not. In other words, *b_jt_* = 1 if the robot can move one step in the respective direction without hitting any walls or obstacles. Otherwise, *b_jt_* = 0.

After computing (Equation 5), we use Equation (6) to bias the initial distribution and compute the probabilities for sampling the next action to be performed. The bias, which we call motion bias, increases the probability of actions that are similar to the last action performed and decreases the probability of dissimilar actions, as exemplified in Supplementary Figure S2. The objective is to reduce initial runtimes by increasing the likelihood of repeatedly choosing similar actions, thus avoiding trajectories such as constantly moving back and forth.


(6) $$\begin{array}{l} {\pi^{\prime }}_{jt}=\frac{{b^{\prime }}_{jt}\pi_{jt}}{\sum_{k}{b^{\prime }}_{kt}\pi_{kt}}\; \end{array}$$

Where

- $\pi_{jt}^{'}$ is the probability of performing action *j* at time *t* after adding the motion bias.
- $b_{jt}^{'}$ is the motion bias for action *j* at time *t* calculated as $b_{jt}^{'}$ = *B_T_*[*j*−*a*_*t*−1_], where *B_T_* is a circular array of predefined weights for trial *T* given by Equation (7), and *a*_*t*−1_ is the action performed during the previous cycle.

As the number of trials increases, we reduce the magnitude of the bias incorporated in the action selection process to exploit the solutions found by the reinforcement learning algorithm. If the bias is not reduced, the model takes longer to start choosing the policy learned by the reinforcement learning algorithm, and the policy may converge prematurely. To reduce the bias, we interpolated an array of predefined weights with a uniform distribution at the start of each trial. The interpolation is done so that, as trials go by, the initial array exponentially decays to a uniform distribution according to Equation (7). Note that as the weights become uniform, the biased distribution $\pi_{jt}^{\prime }$ resulting from Equation (6) becomes more similar to the unbiased distribution π_*jt*_. The predefined weights and the exponential decay rate were constant across all simulations and were empirically chosen to decrease initial runtimes and to prevent the policy from converging prematurely (leading to longer final trial paths).


(7) $$\begin{array}{l} B_{T}[j]=u+\nu^{T}(B_{0}[j]-u) \end{array}$$

Where

- *B_T_* is the circular array of biases for trial *T*. The array exponentially decays to a uniform distribution.
- *u* is a constant (set to 8 ^− 1^) representing the uniform distribution.
- *B*_0_ is the circular array of biases used in the first trial. The array is set so that *B*_0_[0] = 0.83, *B*_0_[1] = *B*_0_[−1] = 0.06, and *B*_0_[*j*] = 0.01 for all other *j*.
- ν is a parameter (set to 2 ^− 1/50^) that controls the array's decay rate.

#### 3.3.5. Eligibility traces

We use eligibility traces to improve the algorithm's efficiency (Sutton and Barto, 2018). As opposed to updating one state at a time, eligibility traces keep track of previously visited states and assign rewards to all of them based on how long ago they were active. Our model's eligibility traces for the critic and the actor are computed according to Equations (8) and (9).

As in Scleidorovich et al. (2020), the equation for the critic (Equation 8) is an adaptation from Llofriu et al. (2019) to normalized radial basis functions, but here, we update the mechanism that deals with very small traces. This mechanism reduces the number of computations per cycle by setting very small traces to 0. The original model sets to 0 all traces that are smaller than constant. Instead, this model introduces a counter for each cell that keeps track of the last time it was active. Then, using the counters, traces are set to 0 when their respective cell has not been active for a given number of cycles.

As opposed to the critic, we replaced the actor's traces from Scleidorovich et al. (2020) with Equation (9). The new equation is an adaptation of the traces for actor-critic algorithms (as defined in Sutton and Barto, 2018) to our implementation of the actor. As for the critic, we used the counters to set very small traces to 0.


(8) $$\begin{array}{l} z_{it}=\{\begin{array}{l} 0\;c_{it}>C^{V}\\ max\{\lambda^{V}z_{i,t-1},P_{it}\}\text{ otherwise } \end{array}\; \end{array}$$

(9) $$\begin{array}{l} z_{ijt}=\;\{\begin{array}{l} 0\;c_{it}>C^{Q}\\ \lambda^{Q}z_{ij,t-1}+(\delta_{a_{t}}^{j}-\pi_{jt})P_{it}\text{ otherwise} \end{array}\; \end{array}$$

Where

- *z_ijt_* and *z_it_* are the traces associated with place cell *i*, action *j*, at time *t* for the actor and critic, respectively.
- λ*^Q^* and λ*^V^* are the decay rates for the actor and critic, respectively. For our experiments, we set λ*^Q^* = λ*^V^*, and all experiments were performed with and without traces (decay rates were set to 0.7 and 0, respectively).
- $\delta_{a_{t}}^{j}$ is the Kronecker delta function that takes the value of 1 if *a_t_* = *j* and 0 otherwise.
- *c_it_* is a counter that keeps track of the number of cycles passed since the last time cell *i* was active. The counter is set to 0 if *P_it_*>0, or else it is set to *c*_*i,t*−1_+1.
- *C^V^* and *C^Q^* are constant parameters (set to $\frac{\ln\;0.0001}{\ln\;\lambda^{V}}$ and $\frac{\ln\;0.0001}{\ln\;\lambda^{Q}}$, respectively) that regulate how many cycles can a trace be active before resetting it to 0. Note that when traces are 0, the constants also become 0.

#### 3.3.6. RL error and learning rule

To update the learning weights associated with each place cell for both the actor and the critic, we use the actor-critic learning rule using semi-gradient descent and the 1-step return bootstrap error (Sutton and Barto, 2018). The formulas for the update are shown in Equations (10)–(13). Equation (10) shows how to compute the bootstrap (i.e., the new approximation of the state value computed from the old approximation and the new data), Equation (11) shows the reinforcement learning error, and Equations (12) and (13) show the update rules for the critic and actor, respectively.


(10) $$\begin{array}{l} {\text{V}}_{t}^{\prime }=\{\begin{array}{l} r_{t}\text{ if terminal state}\\ r_{t}+\gamma \sum_{i}P_{it}V_{i,t-1}\text{ otherwise} \end{array} \end{array}$$

(11) $$\begin{array}{l} \delta_{t}={V^{\prime }}_{t}-V_{t-1} \end{array}$$

(12) $$\begin{array}{l} V_{it}=V_{i,t-1}+\alpha^{V}\delta_{t}z_{i,t-1} \end{array}$$

(13) $$\begin{array}{l} Q_{ijt}=V_{ij,t-1}+\alpha^{Q}\delta_{t}z_{ij,t-1} \end{array}$$

Where

- $V_{t}^{'}$ is the 1-step return bootstrap.
- *r_t_* is the reward received at time *t*.
- γ is the discount factor (set to 0.95).
- *V*_*i,t*−1_ is the value associated with place cell *i* computed at time *t*−1.
- δ*_t_* is the reinforcement learning error computed at time *t*.
- *V_t_*_−1_ is the state value computed at time *t* − 1.
- *z_i,t_*_−1_ and *z_ij,t_*_−1_ are the eligibility traces computed at time *t*−1 for both the critic and actor, respectively.
- α*^V^* and α*^Q^* are the learning rates (both set to 0.4) for the critic and actor, respectively.

### 3.4. Place field distributions

**Figure 3:**
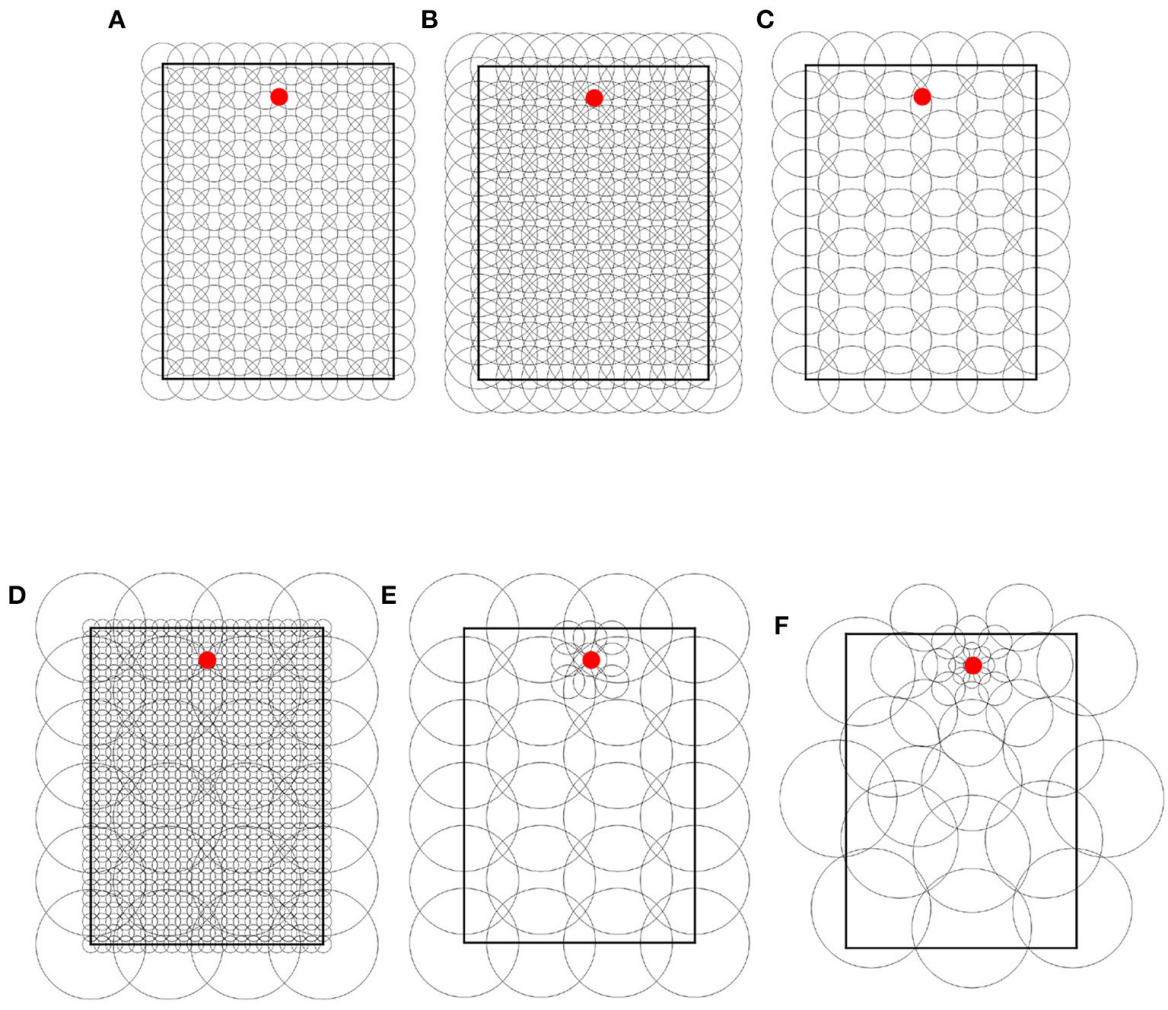
Place cell distribution type samples. **(A)** Uniform single-scale, 20 cm cells, 14 x 10 grid. **(B)** Uniform single-scale, 32 cm cells, 14 x 10 grid. **(C)** Uniform single-scale, 32 cm cells, 9 x 6 grid. **(D)** Uniform multi-scale. **(E)** Locally uniform multi-scale. **(F)** Non-uniform multi-scale. In all samples, the red dots indicate the feeders.

Throughout the experiments, we used 4 types of place field distributions. Each type is illustrated in Figure 3 and described in the rest of this section.

**Uniform single-scale distributions** (Figures 3A–C) cover the entire maze by arranging place fields of identical size over a single rectangular grid with identical distances between columns and rows. To cover the maze, the corners of the grids coincide with the corners of the mazes. Uniform layers were used to assess how obstacles affect different field sizes and to assess optimal cell numbers based on the number of obstacles and place field size.

**Uniform multi-scale distributions** (Figure 3D) cover the maze by combining multiple uniform layers, each covering the entire maze. This distribution type was used to assess whether a reinforcement learning algorithm would give preference to small or large fields based on the distance to obstacles.

**Locally uniform multi-scale distributions** (Figure 3E) cover the maze by combining uniform layers whose corners do not necessarily coincide with those of the maze. Contrary to uniform multi-scale distributions, each sublayer in a locally uniform distribution may cover a small portion of the maze. This distribution type was used to assess whether the results from uniform distributions could be improved by adding additional place cells at specific locations, namely around the goal and near obstacles.

**Non-uniform multi-scale distributions** (Figure 3F) cover the maze by placing place fields of different sizes anywhere on the maze, i.e., they are neither restricted in size nor to a grid in space. Non-uniform layers were created manually based on the hypothesis that small scales are helpful in areas where the policy changes rapidly over space (i.e., near obstacle corners and goals) and vice versa. This distribution type was used to show the advantages of adapting the place field representation (field sizes and positions) to the specific environment.

### 3.5. Evaluation metrics

To evaluate the model, we define the following metrics: **“extra steps ratio,” “learning time,”** and **“scale contribution.”** The first two metrics analyze how well and how fast the agents learn the task, while the latter measures how relevant a scale is for solving a task. They are described next.

#### 3.5.1. Extra steps ratio-path optimality

In Equation (14), we define the metric **“extra steps ratio”** to assess the optimality of the paths learned by the agents. The metric measures the number of extra steps taken to complete a trial beyond the shortest path's length. The concept is illustrated in Supplementary Figure S3. To calculate the metric, Equation (14) first subtracts the minimum number of actions required to complete a trial from the number of actions performed by the rat. Results are then normalized to make them independent from the shortest path's length. As a result, the metric can be thought of as the number of extra steps taken per required step.


(14) $$\begin{array}{l} e_{T}=\;\frac{A_{T}-M\;}{M} \end{array}$$

Where

- *e_T_* is the optimality ratio in trial *T* for a given rat
- *A_T_* is the number of actions performed by the agent during trial *T*
- *M* is the minimum number of actions required to reach the goal in the respective maze

Note that although, in theory, extra step ratios should always be greater or equal to 0, results may be negative as *M* in Equation (14) is only an approximation of the shortest path, calculated using the A-star algorithm (Hart et al., 1968) by discretizing space into a 1 mm square grid. As a result, ratios may be smaller than 0 if the reinforcement learning algorithm finds a better solution than the A-star algorithm.

#### 3.5.2. Learning time

We define the metric **“learning time”** in Equation (15) to measure how fast each agent learns. The metric measures the number of trials that an agent requires to reach a given extra steps ratio for the first time. The concept is illustrated in Supplementary Figure S3. Note that with this definition, learning times may vary greatly depending on the chosen threshold, and there is no guarantee that the extra steps ratio will not increase at a later trial. Nonetheless, the objective of defining the learning time in this way is to assess the initial behavior of the curve “extra steps ratio vs. trial” while ignoring its asymptotic behavior.


(15) $$\begin{array}{l} l\text{ = }arg{min}_{T}{(\{e}_{T}\;<E\}) \end{array}$$

Where

- *l* is the learning time of a given rat, which we define as the first trial in which the extra steps ratio is below a given threshold
- *e_T_* is the rat's extra steps ratio on trial *T*
- *E* is the chosen constant threshold (set to 1)

#### 3.5.3. Scale contributions

Scale contributions assess the involvement of each scale in solving the task. We propose two metrics which we term **“action contribution”** and **“value contribution.”** Both metrics are measured after the final trial of each simulated rat.

The value contribution of a scale is defined in Equation (16). The metric measures the magnitude of the state value contributed by the cells of the respective scale (the numerator in the equation). The magnitude is measured as a percentage of the total state value function (the denominator). Since the quotient depends on the position where it is measured, results average multiple locations (the set *X*). An alternative way of thinking about the metric is that it measures how much the state value function would change by deactivating (i.e., not using) the given scale.


(16) $$\begin{array}{l} C_{sX}^{V}=\frac{1}{\left|X\right|}\sum_{\vec{x}\in X}\frac{\left|\sum_{i:r_{i}=s}V_{i}P_{i}(\vec{x}))\right|}{\left|\sum_{i}V_{i}P_{i}(\vec{x}))\right|} \end{array}$$

Where

- $c_{sX}^{V}$ is the value contribution of scale *s* for a given agent measured on the set of positions *X*
- |·| denotes either set cardinality or absolute values
- *r_i_* is the radius of place cell *i*
- $P_{i}(\vec{x})$ is the activation of place cell *i* as defined by Equation (2) but for position $\vec{x}$ rather than ${\vec{x}}_{t}$
- *V_i_* is the resulting state value associated with place cell *i* after the final trial of the given rat.

The action contribution of a scale is defined by Equation (17) and is very similar to the value contribution. As opposed to the state value function that defines a single value per state, the action value function defines a vector of values per state (one for each action). Thus, the only difference between both equations is the use of the vector norms (rather than the absolute values) to measure the contribution.


(17) $$\begin{array}{l} C_{sX}^{A}=\frac{1}{\left|X\right|}\sum_{\vec{x}\in X}\frac{\left||\sum_{i:r_{i}=s}{\vec{Q}}_{i}P_{i}(\vec{x}))|\right|}{\left||\sum_{i}{\vec{Q}}_{i}P_{i}(\vec{x}))|\right|} \end{array}$$

Where

- $c_{sX}^{A}$ is the action contribution of scale *s* for a given agent measured on the set of positions *X*
- |·| denotes set cardinality
- ||·|| denotes the Euclidean norm
- *r_i_* is the radius of place cell *i*
- $P_{i}(\vec{x})$ is the activation of place cell *i* as defined by Equation (2) but for position $\vec{x}$ rather than ${\vec{x}}_{t}$
- ${\vec{Q}}_{i}$ is the resulting vector of action values associated with place cell *i* after the final trial of the given rat.

Note that in the definitions above, both equations depend on the set *X* where the metrics are evaluated. Our experiments consider different sets, but the details are left to the respective sections.

## 4. Experiments and results

In total, we performed 4 experiments with the model using the SCS simulator^1^. The code for this project can be found on our lab's GitHub repository^2^. Parallel simulations were performed using CIRCE, which is one of University of South Florida's computer clusters^3^.

The experiments described in this section present variations in place cell representations adapted to different environments. Results are analyzed in terms of the previously described evaluation metrics. We start by analyzing single-scale uniform layers and then non-uniform multi-scale distributions. Note that although all experiments were performed with and without eligibility traces (with decay rates set to 0.7 and 0, respectively), results were similar for both settings, and thus we only report results without traces unless otherwise stated.

### 4.1. Experiment 1-field size vs. obstacles

#### 4.1.1. Objective

The goal of experiment 1 is to analyze the effects of single-scale place field representations, i.e., place field sizes, on different obstacle configurations. We evaluate learning times, path optimalities, and the optimal numbers of cells, for single-scale uniform distributions on variations in the number of obstacles. We hypothesize that, 1) as the number of obstacles increases, both learning times and extra step ratios will also increase, 2) higher cell numbers will result in slower learning but will reach better results (lower extra step ratios during final trials), 3) optimal distributions will require more cells at higher obstacle densities, and 4) compared to larger place fields, the results obtained with smaller fields will be more robust against changes in the number of obstacles.

#### 4.1.2. Parameter configuration

Experiment 1 evaluates the model using 97 uniform single-scale place cell distributions in 61 mazes with 7 different numbers of obstacles.

**The number of obstacles** used in the mazes for this experiment varied from 0 to 60 in increments of 10. For each non-zero obstacle number, 10 mazes were randomly generated by placing obstacles in different configurations as described in Section 1. Sample mazes are illustrated in Figure 1B.

**Single-scale uniform distributions** involved variations in the number of cells and the field sizes. Field radii ranged from 4 to 56 cm in increments of 4 cm. The total number of cells was controlled by modifying the number of columns in the uniform grid. Columns varied between 5 and 40 in increments of 5, generating distributions between 35 and 2,200 cells. Additionally, we tested the minimal coverage distribution (MCD) of each scale corresponding to the least number of cells necessary to cover the maze. Figures 3A–C illustrate 3 sample distributions. See Supplementary Section 2.1 for a full description of all uniform distributions used.

To generate statistical data, we simulated 135,800 agents in total with 100 agents per group, i.e., 100 agents per distribution, per number of obstacles, per trace. For each condition with non-zero number of obstacles, 10 agents were simulated for each obstacle configuration to avoid biases introduced by any specific configuration.

#### 4.1.3. Results-learning time

**Figure 4:**
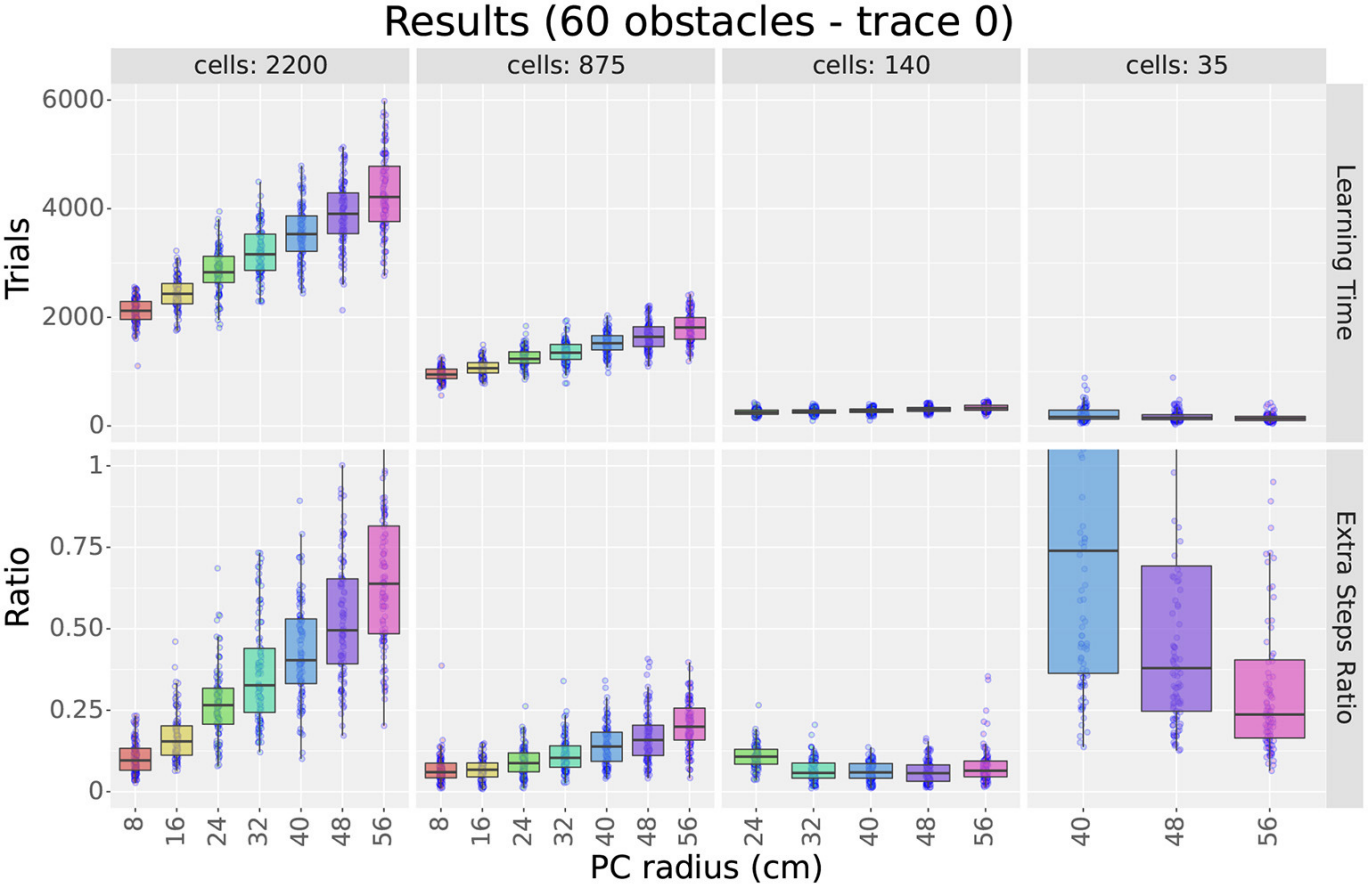
First experiment results as a function of the field size. The figure compares the learning time **(top row)** and extra step ratio **(bottom row)** box plots of seven field sizes for different cell numbers **(columns)** when using 60 obstacles. For 24 and 140 cells, some field sizes are missing as the resulting layers would not cover the entire maze.

**Figure 5:**
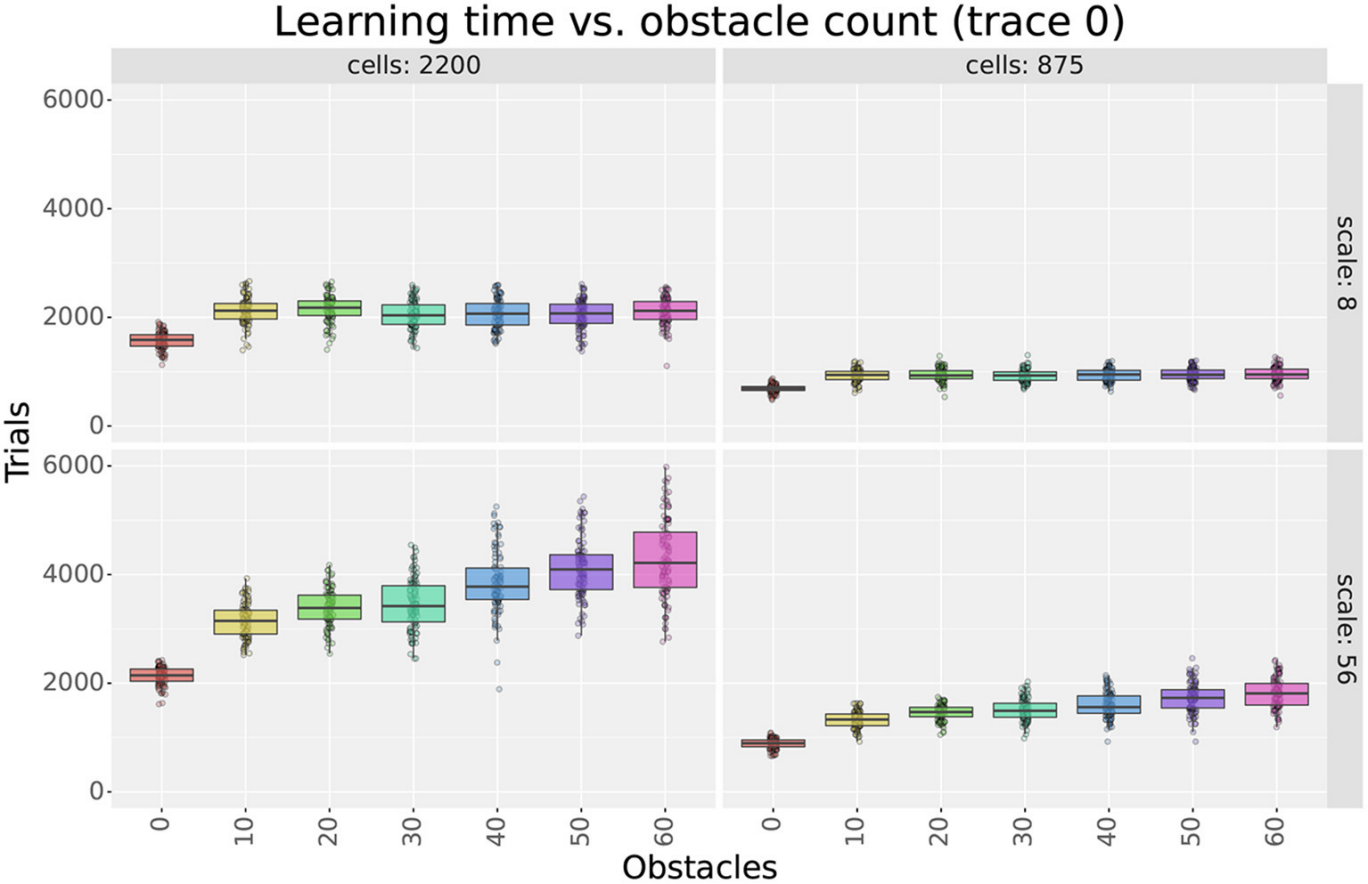
Learning time as a function of the number of obstacles in the first experiment. The figure compares the learning time box plots of seven obstacle numbers for different field sizes **(columns)** and cell numbers **(rows)**.

Figures 4 (top row) and 5 show sample learning times achieved by the agents using the single-scale uniform distributions. Figure 4 illustrates the effects of varying the number of cells and the scale, while Figure 5 focuses on the effects of changing the number of obstacles. Only a subset of the results are shown as the experiment compared 1,358 parameter configurations. More detailed results are shown in Supplementary Figures S5, S6.

As observed in Figure 4, results show that increasing either cell numbers or field sizes increased learning times. This was true in all cases except when using 35 cells or less in obstacle-rich environments. In such circumstances, increasing either the scale or the number of cells reduced learning times. Based on the results discussed in Section 4.1.4, we attribute these exceptions to difficulties in learning when using very sparse representations. In other words, it is not that very sparse representations yield slower learning but that they are unable to learn efficient paths in cluttered environments.

When comparing factors, one key observation is that the number of cells was more relevant than the field size in determining learning times. This is best observed in Figure 4, where the lowest learning times were obtained by reducing the number of cells to 140. Although reducing field sizes also led to shorter learning times, the reduction due to the number of cells was a larger order of magnitude. Importantly, this observation provides motivation to reduce the number of cells when constructing non-uniform layers and thus reduce learning time.

Considering obstacles, our first observation is that adding obstacles made more evident learning time differences between different scales and cell numbers. For example, when using 2,200 cells in Figure 5, the difference in learning time between scales 8 and 56 was about 250 trials in empty mazes but about 2,000 in mazes with 60 obstacles. This observation highlights the importance of testing the model in cluttered environments and suggests that differences in dorsal and ventral place cells should be assessed in complex rather than simple environments.

Our second observation regarding obstacles is that the learning times of larger scales were more affected by the number of obstacles than smaller scales. Figure 5 shows that learning times consistently increased when switching from 0 to 10 obstacles, but results varied for higher obstacle numbers. For smaller scales, such as scale 8, learning times remained unchanged. For larger scales, such as scale 56, learning times increased at a rate proportional to the field size. Although the number of cells also modulated the rate, differences between scales were observed regardless of the number of cells. Consequently, results suggest that smaller fields are better suited for cluttered environments than larger fields.

See Supplementary Section 2.2 for a discussion of whether longer learning times due to obstacles could be explained by longer exploration times during initial trials.

#### 4.1.4. Results-extra steps ratio

The bottom row of Figure 4 shows sample extra step ratios achieved by the agents during the final trial of the experiment. The figure illustrates the effects of varying the number of cells and the scale. As for the learning times, the figure only show a subset of the results due to the large number of parameter configurations tested. Additionally, although we plotted the extra step ratios vs. the number of obstacles, results are very similar to its learning time counterpart. Thus, we will assess the results but omit the extra figure. More detailed results are shown in Supplementary Figures S8, S9.

Although we hypothesized that higher place cell numbers would lead to better extra step ratios during final trials, Figure 4 shows this was not the case. Instead, optimal cell numbers varied according to the scale and the number of obstacles (see Supplementary Section 2.3 for details). With few exceptions, the best results were achieved using 560 cells or less. Increasing the number of cells above the optimum gradually increased the extra step ratios. On the other hand, reducing the number led to sharp deterioration. This is best exemplified by scale 40 in Figure 4. In mazes with 60 obstacles, scale 40 reached a peak performance of about 0.1 extra steps ratio at 140 cells. Although adding cells slowly increased the ratio, removing cells increased it quickly to its worst value reached at 35 cells.

We attribute the existence of a ‘sweet spot' in the number of cells to the following two factors. 1) The fast deterioration of extra step ratios when using very few cells suggests that very sparse distributions have difficulties learning the optimal paths. This is further evidenced by noting that sparse distributions were more sensitive to the number of obstacles than distributions with more cells (see Supplementary Figure S8). 2) Longer learning times can explain the increased extra step ratios when using more cells. Taking scale 40 from Figure 4 as an example, if we assume that all distributions with more than 140 cells can learn the optimal paths, the only difference would be the time it takes to learn them. Results from Section 4.1.3 showed that increasing the number of cells led to longer learning times. Thus, if a layer has not yet finished learning, adding cells would lead to larger final extra step ratios. Combined, these observations imply that larger place cell numbers allow for better representation and learning of the paths (i.e., shorter learning times and extra step ratios), but adding more cells than required slows learning and increases final extra step ratios. These observations are important because they suggest that optimal distributions should use the least number of cells required to solve a maze.

As observed in Figure 4, increasing field sizes led to results that varied according to the number of cells. When using 560 cells or more, final extra step ratios increased proportionally to the field size, with the best results achieved by the smallest scales. When using 35 cells or less, results were inverted, with larger scales outperforming smaller scales. For intermediate cell numbers, results varied between the two extremes, typically achieving the best results within the 3 smallest scales. Similar to the learning times, adding obstacles also increased the difference between scales. Also, note that distributions using very few cells were the most affected by adding obstacles, abruptly increasing their extra step ratios (see Supplementary Figure S8).

Lastly, when assessing the effect of the number of obstacles, results were very similar to those obtained with the learning times. The extra step ratios increased for large scales but remained unchanged for small scales. The only difference with the results shown for the learning times is that there is no jump in extra step ratios between the 0 and 10 obstacle conditions. Due to the similarity between plots, we believe that the larger extra step ratios were caused by the longer learning times and not by higher difficulty representing the optimal paths.

### 4.2. Experiment 2-scale contribution

#### 4.2.1. Objective

For the second experiment, we want to assess the “importance” of each scale for encoding the final policy in a uniform multi-scale model. By “importance,” we mean “how much does the final policy and value functions depend on a given scale?.” To answer the question, we defined metrics “action contribution” and “value contribution” in Section 3.5.3. Each metric quantifies how much the state and action value functions would change when deactivating (not using) a given scale. The objective of the experiment is to show that smaller scales are more relevant for encoding areas near decision points, while larger scales are more relevant for encoding open spaces far from decision points.

Given the contribution metrics, we hypothesize that: 1) the contribution of smaller scales will increase near subgoals (i.e., places where the agent must change directions) and decrease farther away, 2) the contribution of larger scales will decrease near subgoals and increase farther away, and 3) as the number of obstacles increases, the number of decision points will also increase, leading to the same prediction as in 1. These hypotheses are based on the idea that larger fields are useful to reduce the number of cells required, while smaller scales are useful to encode details.

#### 4.2.2. Parameter configuration

For experiment 2, we assessed the contributions of all scales in a single uniform multi-scale distribution. The distribution is illustrated in Figure 3D and combines the minimal coverage distributions of scales 4, 16, and 52 from experiment 1.

Experiment 2 was performed in all mazes from experiment 1 except for the empty maze. As a result, we simulated 10 agents per maze in 60 obstacle-rich environments or, alternatively, 100 agents per obstacle number.

For each rat, contribution metrics were evaluated over two sets of positions: “All positions” from a rat's final trial, and “Turns only,” final trial positions where the agent made a turn (i.e., where it changed directions). The objectives for choosing these sets were two-fold. First, we want to avoid measuring the contribution in areas that are irrelevant to the final trial. Such areas may not be optimized by the algorithm and thus may contain irrelevant information. Second, the set of turns should closely reflect the policy's decision points as agents explore little after convergence. As a result, we expect that contributions in the set of turns will increase for smaller scales and decrease for larger scales when compared to the set of all positions.

#### 4.2.3. Results

**Figure 6:**
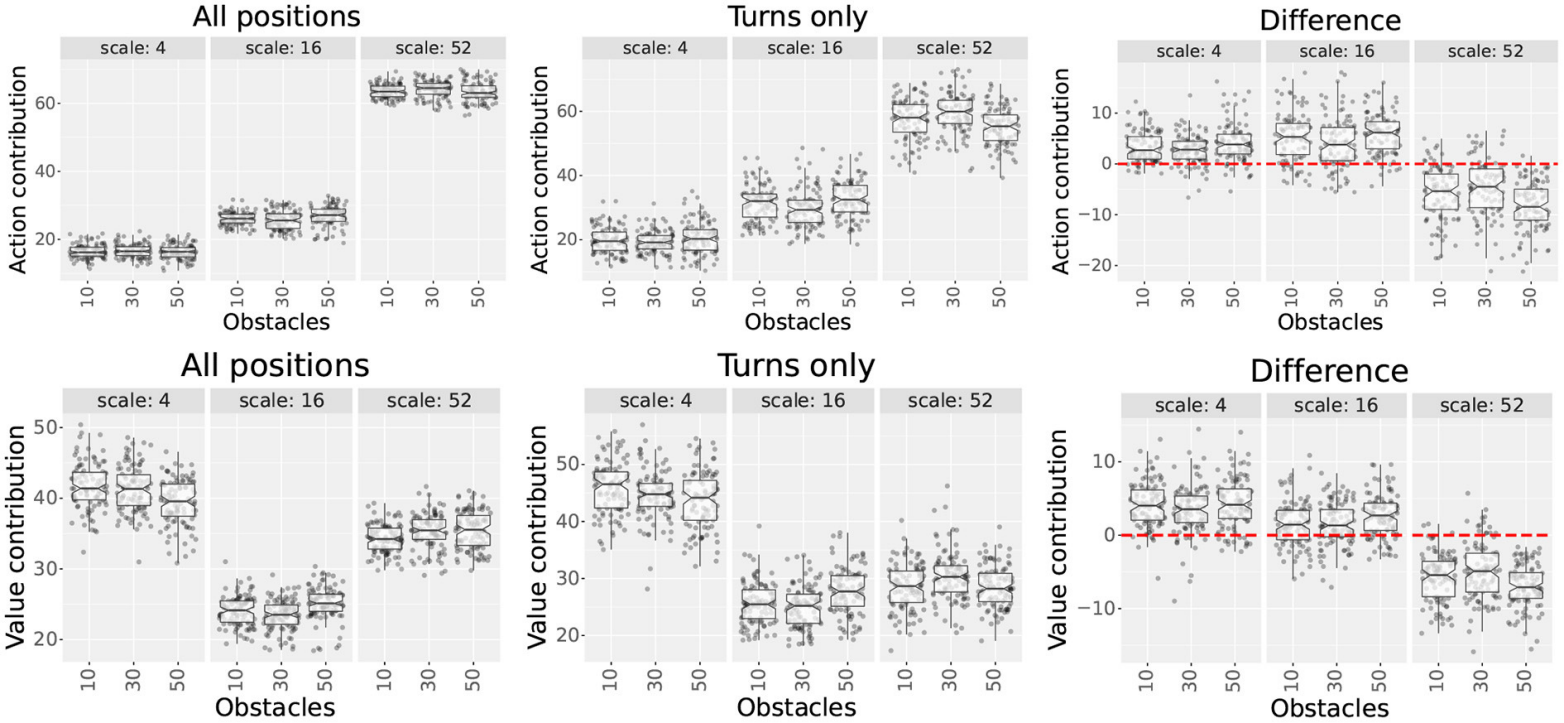
Scale contributions of the multi-scale uniform distribution used in experiment 2. The plots show each scale's action and value contributions (first and second rows, respectively) for three obstacle numbers. The first and second columns show the contributions measured in the set of all positions and in the set of turns, respectively. The third column shows the difference (subtraction) between the results of the second and first columns. The red dotted line highlights the 0 y-coordinate.

The results of the experiment are shown in Figure 6. The figure shows the action and value contributions for each scale for sample obstacle numbers. The plots in the first two columns show the results in the set of all positions and the set of turns, respectively. The third column shows the subtraction between the contributions of both sets, highlighting their difference. Positive numbers indicate that the contribution was higher in the set of turns than in the set of all positions and vice versa.

Results show that the contribution differences between both position sets varied across scales. As hypothesized, contributions increased for smaller scales (4 and 16) and decreased for larger scales (52), suggesting that smaller scales become more relevant near decision points and vice versa. This pattern was consistent for both metrics for all obstacle numbers. Additionally, although we expected that the contributions of scale 4 would increase more than scale 16, this was only true for the value contribution metric.

When assessing the effect of increasing the number of obstacles, there were no consistent increases or decreases in the contribution of smaller and larger scales. Since this contradicted our hypothesis, we decided to investigate further.

First, we performed Kruskal Wallis tests for each scale and position set to assess statistical differences between the number of obstacles. After confirming statistical differences (*p* < 0.05), we followed the results with Dunn tests using Bonferroni corrections. Results from the Dunn tests showed that most distributions were not significantly different. When significant differences were present, we did not find any patterns except for the following. Value contributions for scales 4 and 16 could be partitioned into one group with 40 or fewer obstacles and another with 50 or more. For that partition, differences were significant across groups but not within groups.

Since most differences between obstacle numbers were not significant, we plotted the optimal path lengths, the extra step ratios during the final trial, and the number of turns performed by the agents. Our hypothesis assumed that adding more obstacles would increase the number of turns made by the agents, but the results indicated this was not the case. Supplementary Figure S11 shows that the length of the optimal paths is the same for 58 out of the 60 mazes. Also, for mazes with 50 and 60 obstacles that have significantly higher numbers of turns (*p* < 0.05 in Dunn test), the extra step ratios were also significantly higher. Taking all into account, rather than the optimal policies requiring more turns, our results suggest that the increase in turns is due to the multi-scale model having higher difficulties learning optimal policies in these obstacle-rich environments.

### 4.3. Experiment 3-locally uniform layers

#### 4.3.1. Objective

In the third experiment, we wanted to assess whether learning times and extra step ratios could be jointly optimized using locally uniform layers. The idea is to cover the maze using large uniform layers to reduce learning times and to add smaller place cells at strategic regions to reduce the extra steps.

Here we hypothesize that adding additional place fields around subgoals can reduce extra step ratios without significantly increasing learning times. The underlying idea is that high place cell densities are not required throughout the whole maze but only in specific regions.

#### 4.3.2. Parameter configuration

Experiment 3 was performed in mazes 0 and 1 (both illustrated in Figure 1B). Maze 0 is an empty maze chosen for its simplicity. In contrast, maze 1 includes two walls that divide the maze in halves and connect them through a small gap, thus generating an extra non-rewarded subgoal. Maze 1 was designed to maximize the difficulty for large scales by creating an area where high precision is required to solve the maze.

To assess our hypothesis, experiment 3 compares single-scale uniform distributions before and after adding two extra layers of place cells. We use the term base layers to refer to the uniform distributions before adding subgoal cells. Base layers include the 10 minimal coverage distributions used in experiment 1 with field sizes between 20 and 56 cm. Distributions using smaller or fewer place cells were excluded as the idea of the base layers is to cover the maze with few larger cells.

**Figure 7:**
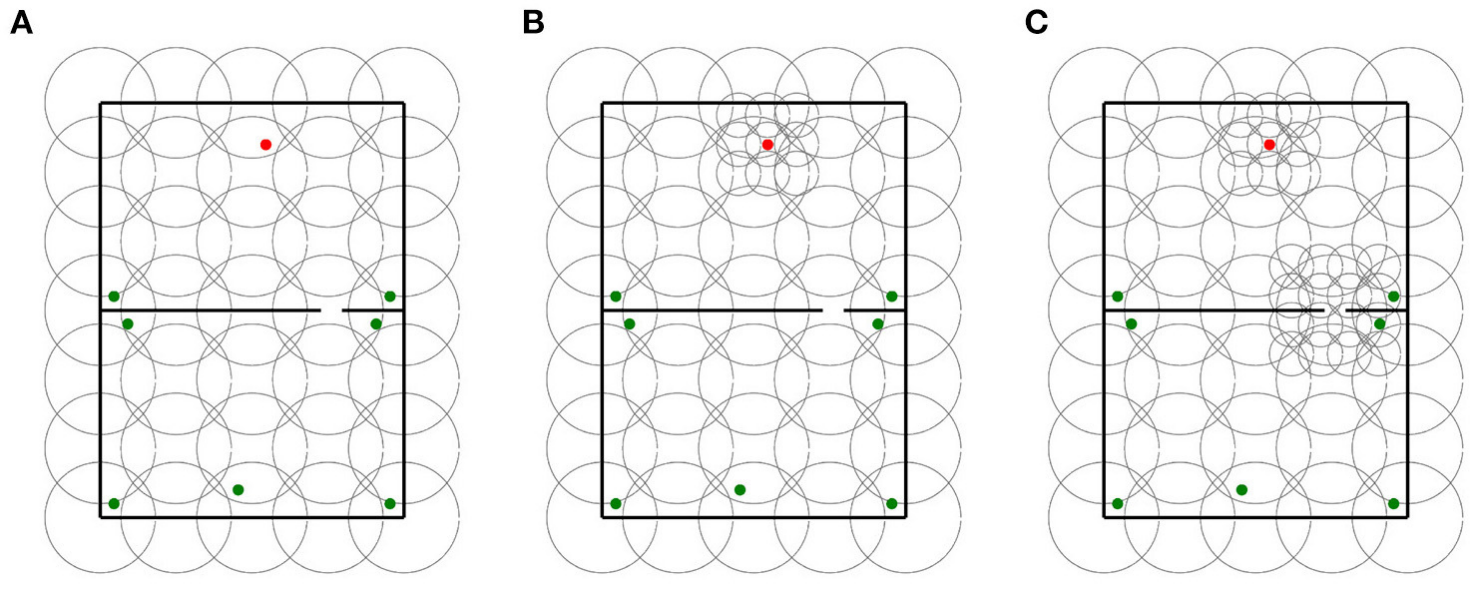
Sample distributions used for in experiment 3. **(A)** Base layer distribution. **(B)** Goal distribution. **(C)** Goal and gap distribution.

For each base layer, two locally uniform distributions were generated. The first locally uniform distribution added a 3 x 3 grid of 16 cm place cells centered around the goal. The second layer added an extra 4 x 4 grid of 16 cm place cells centered around the gap in maze 1. We use the terms “goal distributions” and “goal and gap distributions” to refer to the respective locally uniform distributions. In contrast to base layers and goal distributions that were tested in mazes 0 and 1, goal and gap distributions were only tested in maze 1 as they were designed explicitly for this maze. In total, 30 distributions were assessed in this experiment. Sample distributions of each type are shown in Figure 7.

In total, 100 agents were simulated for each distribution and maze.

#### 4.3.3. Results

**Figure 8:**
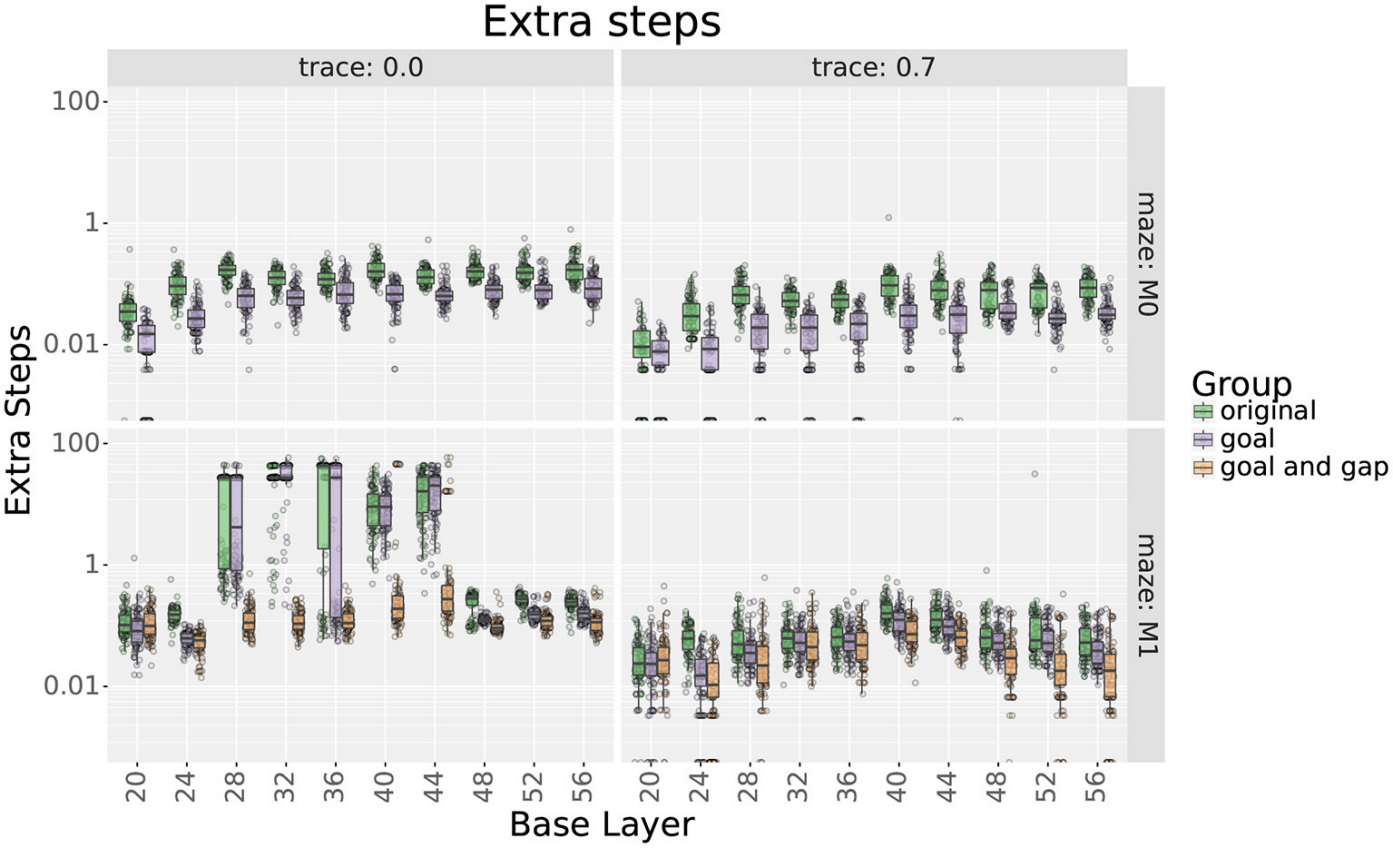
Extra step ratios comparing uniform with their respective locally uniform layers after adding cells at the goal and the gap. Rows show results for mazes 0 and 1, respectively. Columns show results with and without traces, respectively. Note that the y axis uses a logarithmic scale.

**Figure 9:**
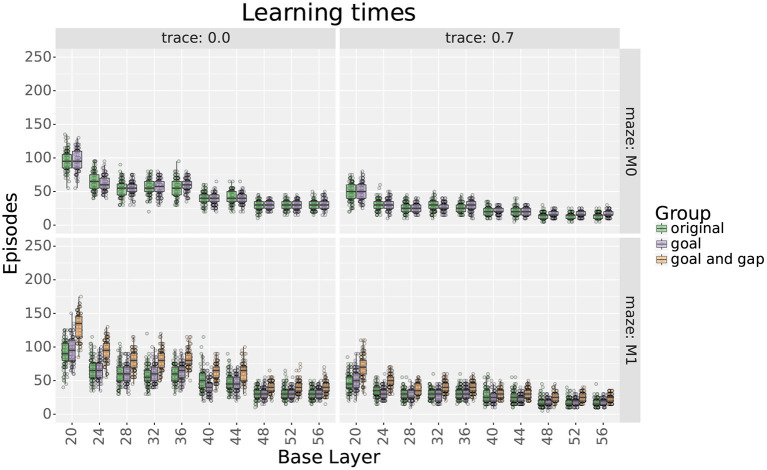
Learning times comparing uniform layers with their respective locally uniform layers after adding cells at the goal and the gap. **Rows** show results for mazes 0 and 1, respectively. **Columns** show results with and without traces, respectively.

Figures 8, 9 show the experiment results, comparing the extra step ratios and learning times before and after adding the additional layers of place cells. For this experiment, results are shown with and without eligibility traces as qualitative differences were observed.

For maze 0, results show that adding 9 place cells around the goal effectively reduced the extra step ratios during the final trial without increasing the learning times. Exceptions were found for scales 20, 36, 48, 52, and 56 only when using traces. For scale 20, adding cells did not improve the extra step ratios as they were already optimal. On the other hand, for scales 36, 48, 52, and 56, extra step ratios were significantly decreased at the expense of longer learning times which took about 10–15 more trials to reach an extra steps ratio of 1.

For maze 1, results were similar, but the main difference was that the agent had trouble learning optimal solutions with most of the original layers, as seen in Figure 8. This was expected as the maze was explicitly designed to be more challenging for larger scales. Note that if we only looked at the results without traces, we could think that mid and large scales were incapable of learning optimal solutions to the maze, but this was not the case, as illustrated by the results using traces. Also, contrary to intuition, the largest scales reached smaller optimality ratios than medium scales. Upon investigation, this is likely due to the automatic placement of cells in uniform layers.

As for maze 0, adding cells around the goal in maze 1 significantly reduced extra step ratios without leading to increased learning times. When not using traces, exceptions were found for scales between 28 and 44 that correspond with the scales that had trouble learning the maze, as seen in Figure 8.

Adding more cells around the gap in maze 1 also decreased final extra step ratios but at the expense of slower learning times (see Figures 8, 9); nevertheless, the increased learning time was still shorter than uniform layers with higher numbers of cells. Exceptions were found only for scales 20 and 24. For scale 24, after adding cells, there was no statistical difference in extra step ratios during the final trial, but the ratio was already near-optimal. On the other hand, although the ratio increased for scale 20, we would argue that this is likely due to the extended learning time as the difference almost disappears when using traces.

Supplementary Section 3 assesses qualitative effects of adding cells around the goal and gap.

### 4.4. Experiment 4-non-uniform distributions

#### 4.4.1. Objective

In the last experiment, we wanted to assess the ability of the model to jointly optimize the number of cells, learning times, and extra step ratios using non-uniform place cell distributions. In particular, we hypothesized that non-uniform distributions could achieve simultaneous optimizations using field sizes proportional to the distance to subgoals (decision points).

Our hypothesis is based on the idea that larger scales are more relevant for encoding areas where the policy changes slowly over space, while smaller scales are more relevant for encoding areas where the policy changes fast. Within information theory (Reza, 1994), this can be intuitively thought of in terms of compression rates and amounts of information. Places where the policy changes slowly have little information and can be encoded using a few larger cells. On the other hand, places where the policy changes fast encode more information and require more cells. Previous experiments suggest that in such regions, place cells should be smaller to reduce learning times and prevent incorrect policy generalizations.

Furthermore, we also suggest that field sizes in optimal distributions should be modulated by the distance to subgoals. Evidence can be found by observing that the rate of change of the optimal policy is indirectly proportional to these distances. The idea is illustrated in Supplementary Figure S13, where the further away from the next subgoal, the less the policy changes within a given region. This can be explained considering that the optimal policy in a circle centered at the next subgoal would move the agent in a straight line (radially) toward the subgoal. Thus, the rate of change would be equivalent to the circle's curvature, which is inversely proportional to the radius (Pressley, 2001) (i.e., to the distance to the subgoal).

#### 4.4.2. Parameter configuration

To assess our hypothesis, we compared the learning time, extra step ratios, and the number of cells of the model using uniform and non-uniform distributions. Uniform distributions included the 14 minimal coverage distributions used in experiment 1.

Non-uniform distributions were manually designed for each maze assuming field sizes should be proportional to the distance to possible subgoals. Possible subgoals include the goal itself and non-convex vertexes in the maze (vertexes whose interior angle is greater than 180 degrees). The presence of non-convex vertexes indicates that not all pairs of points can be connected through a straight line. In such cases, the shortest path between points consists of a polyline passing through any number of non-convex vertexes, thus the reason for considering them possible subgoals. In our mazes, the non-convex vertexes correspond to the corners of the obstacles. As an example, the gap in maze 1 is considered a subgoal since it is close to the corners of the walls. As such, an agent must pass through the gap to move from one half of the maze to the next. Based on these ideas, non-uniform distributions were generated by placing smaller place fields around the goal and obstacle corners and then recursively surrounding them with larger fields.

**Figure 10:**
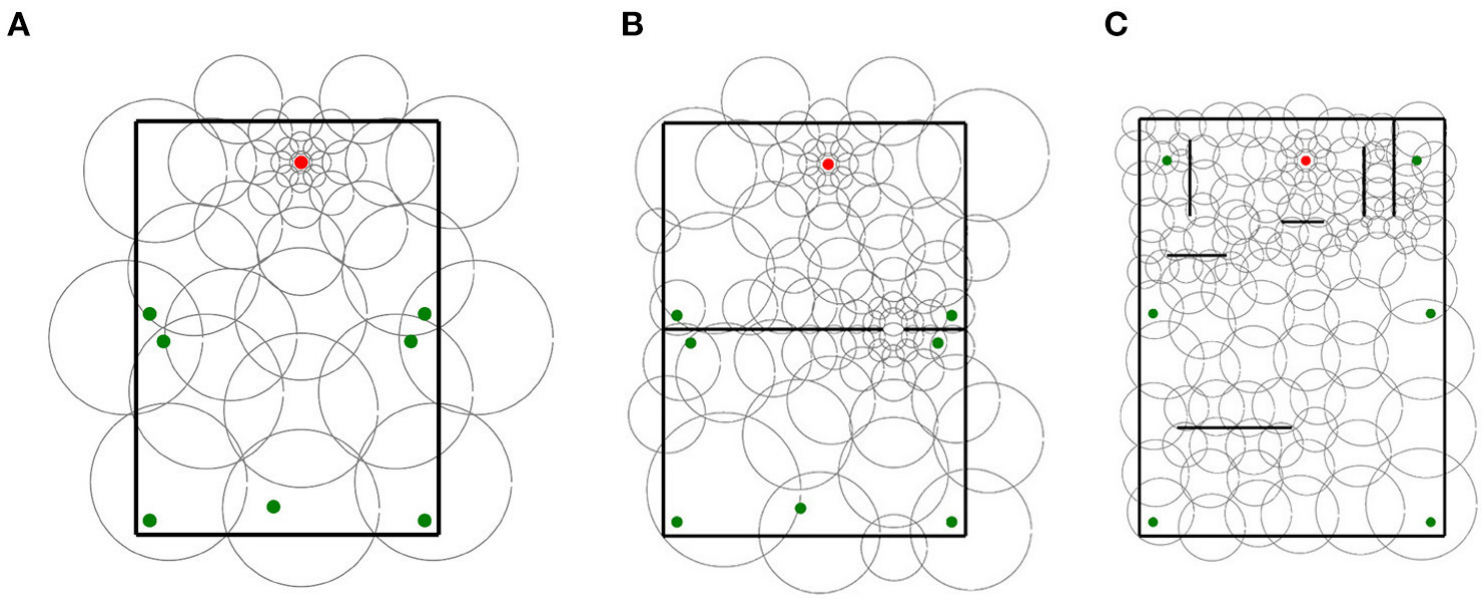
Handmade non-uniform distributions used in experiment 4 for each maze. **(A)** Maze 0, **(B)** Maze 1, and **(C)** Maze 2.

Since non-uniform distributions were manually generated, we only assessed 3 non-uniform layers for 3 specific mazes. Figure 10 illustrates the distributions in their respective mazes. In total, 100 agents were simulated for each distribution and maze.

#### 4.4.3. Results

**Figure 11:**
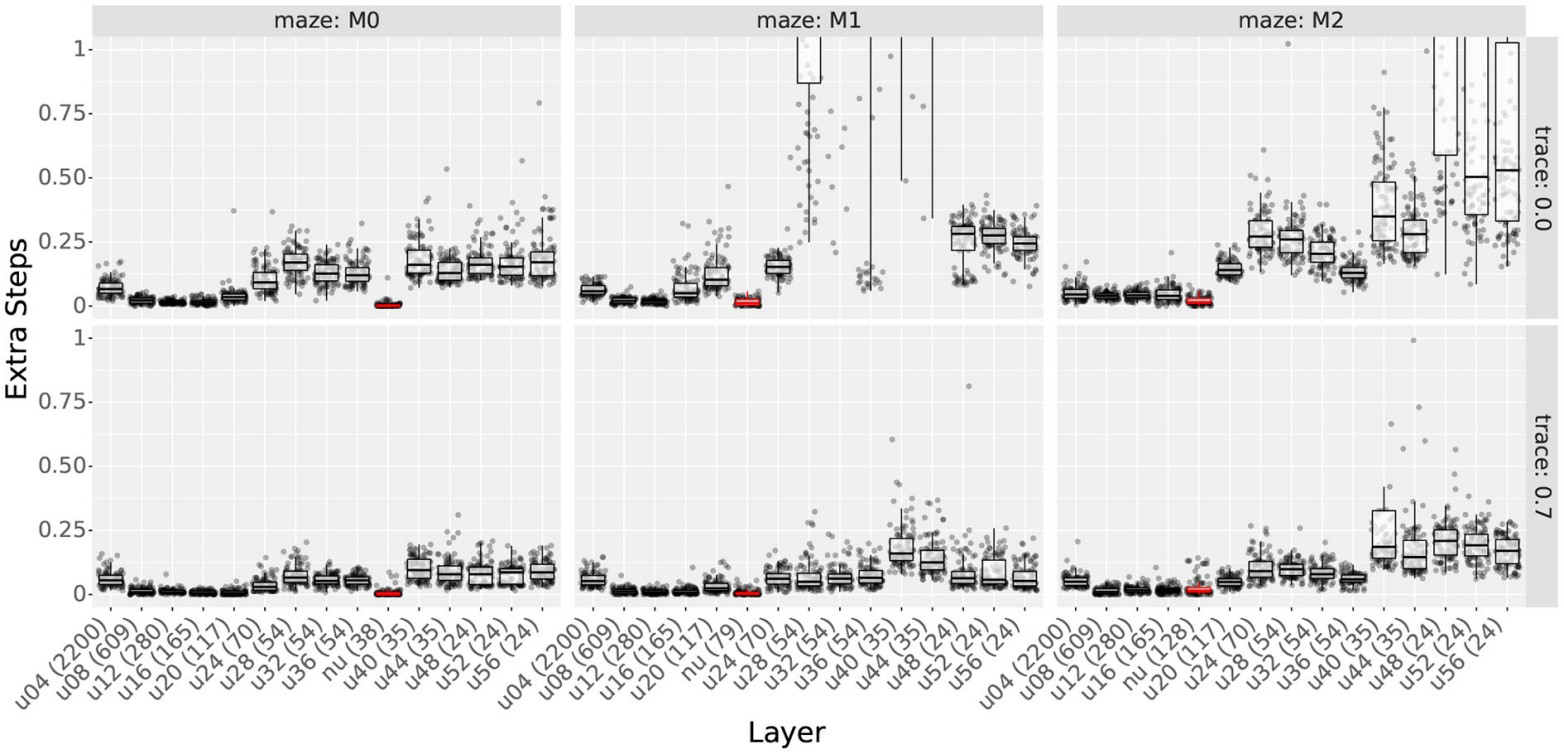
Extra step ratios comparing uniform distributions with non-uniform distributions. **Column** shows the results for mazes 0, 1, and 2. **Rows** show the results with and without traces. Layers are sorted according to the number of cells (shown in parenthesis in each layer's name). Prefixes “u” and “nu” indicate uniform and non-uniform distributions; also, non-uniform distributions are highlighted in red. For uniform distributions, the two digits following the prefix indicate the scale.

**Figure 12:**
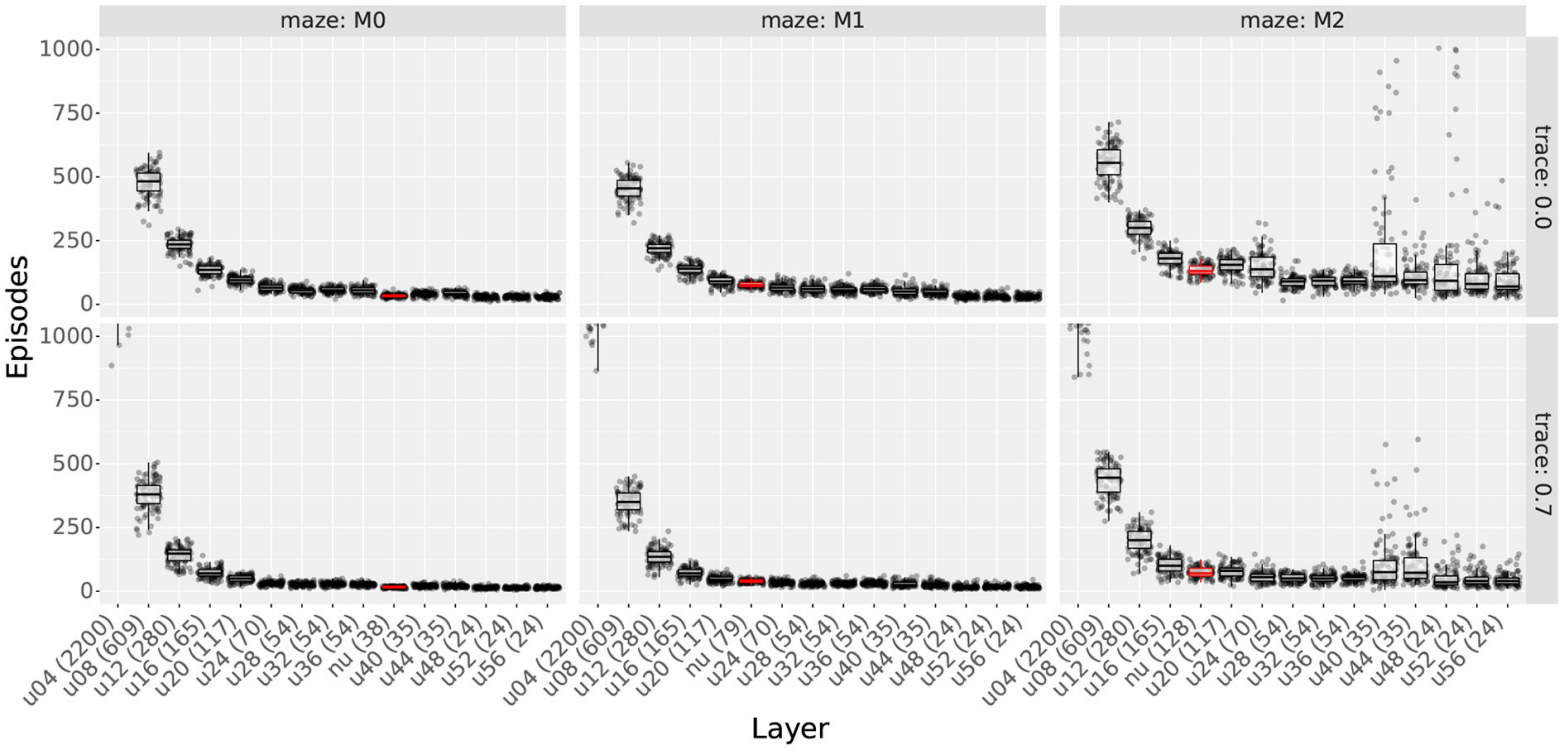
Learning times comparing uniform distributions with non-uniform distributions. **Column** shows the results for mazes 0, 1, and 2. **Rows** show the results with and without traces. Layers are sorted according to the number of cells (shown in parenthesis in each layer's name). Prefixes “u” and “nu” indicate uniform and non-uniform distributions; also, non-uniform distributions are highlighted in red. For uniform distributions, the two digits following the prefix indicate the scale.

Figures 11, 12 show the extra step ratios and learning times, comparing the results of uniform and non-uniform distributions for each maze. As in experiment 3, qualitative differences were observed when using traces. Thus, we included the results with and without traces.

As observed in Figure 11, non-uniform distributions reached the lowest extra step ratios using fewer cells than the best uniform layers of each case. This was true for all mazes and traces. For uniform layers, the best results use at least 117 cells on maze 0 and 165 on mazes 1 and 2. In contrast, non-uniform layers used 38 cells on maze 0, 79 on maze 1, and 128 on maze 2. Note that as the number of obstacles increases, the difference in the number of cells becomes smaller. This is expected as more obstacles imply more areas where higher place cell densities are required. Also, even though by maze 8, the difference in the number of cells is approaching 0, it must be noted that multi-scale distributions were designed following the ideas from Section 4.4.2, and it is likely that optimized distributions would use fewer cells.

When assessing learning times, we again found that the total number of cells is the main factor contributing to the results. This can be observed in Figure 12, where the learning time decreases monotonically with the total number of cells with few exceptions.

Since the resulting learning times can be sorted by the number of cells, not only did non-uniform layers reach the lowest extra step ratios, but also, they did it faster than all uniform layers that achieved similar extra step ratios. To show this, we plotted the extra step ratios vs. trial in Supplementary Figure S14. Although layers with fewer cells had lower learning times, the solutions found by them had higher extra step ratios, and in some cases, they even had issues learning, as previously exemplified by agents getting stuck in maze 1.

## 5. Discussion

In this article, we investigated how to distribute place fields in cluttered environments to simultaneously optimize the learning time and path distance metrics while also reducing the total number of activated place cells. The experiments presented in the paper assessed uniform, non-uniform, single-scale, and multi-scale place field distributions. Results suggest that non-uniform multi-scale place field representations can simultaneously optimize the different metrics by assigning field sizes proportional to their distance to the closest subgoal. As part of the study, we made the following observations when assessing the effects of different numbers of obstacles: 1) When using single-scale distributions, all scales could solve the mazes provided sufficient cells were used. 2) Increasing the number of obstacles led to longer learning times and required higher numbers of cells on average. 3) The results of small fields were more robust against changes in the number of obstacles than the results of large fields. 4) Increasing the number of obstacles, the number of cells, or the field sizes led to longer learning times, but the number of cells was the most significant factor between them. 5) Optimal cell numbers for single-scale uniform models varied according to the maze and field size and were generally achieved using nearly the minimum number of cells to cover the maze for all scales. Using fewer cells destabilized learning and led to longer final paths while using more cells increased learning times also leading to less optimal solutions. A more in-depth discussion of the results is described in the following subsections.

### 5.1. Experiment 1

When assessing optimal numbers of cells, each scale had a “sweet spot” that minimized final extra step ratios. The sweet spot was generally achieved using just enough cells to learn the task. Using fewer cells sharply disrupted the layers' ability to represent paths accurately and destabilized learning. On the other hand, using more cells increased learning times and, as a result, final extra step ratios. For most scales, the best results were achieved using between 140 and 300 cells. Exceptions included very small fields that could, otherwise, not cover the maze. Also, adding obstacles slightly increased the optimal number of cells when averaging all scales.

When comparing the different field sizes, results suggest that smaller scales are better than larger scales at encoding cluttered environments and optimizing extra step ratios. Evidence comes from the following. First, smaller scales generally reached lower extra step ratios than larger scales when using the same number of cells. Second, results for smaller scales were more robust than larger scales when adding obstacles. That is, although adding obstacles increased the learning time and final extra step ratios of all scales, the increase was higher for larger scales than for smaller ones. Increasing the number of cells only made the differences more visible. Additionally, results after adding obstacles remained nearly unchanged for the smallest scales.

As opposed to the extra steps, larger scales were more useful than smaller scales for reducing learning times by encoding large obstacle-free regions. Results showed that the number of cells was the most critical factor in minimizing learning times. As a result, although smaller scales allow for faster learning than larger scales when using the same number of cells, larger scales can reduce the number of cells required to cover an environment and can thus reach shorter learning times.

When combined, results suggest the use of smaller cells to encode obstacle cluttered areas and few larger cells to encode open fields. These predictions go in hand with other computational models that suggest place fields should account for the environment's layout (Gustafson and Daw, 2011; Harland et al., 2021). This is further supported by biological experiments that found some place fields activate or deactivate when obstacles are introduced (Muller and Kubie, 1987; Rivard et al., 2004) and by the discovery of boundary and object vector cells in the brain (Lever et al., 2009; Hoydal et al., 2018) that are thought to drive place cell activity (O' Keefe and Burgess, 1996; Burgess et al., 2000; Hartley et al., 2000).

### 5.2. Experiment 2

Experiment 2 assessed the contributions of different scales in a uniform multi-scale model. As hypothesized, results showed that both the action and value contributions increased near decision points for smaller scales and decreased for larger scales. As a result, the experiment motivates the idea that non-uniform distributions should use smaller scales near decision points and larger scales when far.

When adding obstacles, we did not observe significant differences. Although this contradicts our original predictions, our premise was that more obstacles meant more turns (decision points). To our surprise, this was not the case, as there were no statistical differences in the number of turns made by the robot in the different mazes. As a result, we found that randomly placing 10 to 60 small obstacles in a maze did not significantly increase the difficulty of representing the final policy and value functions.

### 5.3. Experiments 3 and 4

Experiments 3 and 4 assessed two types of non-uniform place field distributions. The objective was to assess whether the distributions could jointly optimize the number of cells, the learning time, and the final extra steps ratio.

Experiment 3 used locally uniform layers and acted as a proof of concept. The experiment assessed whether the final extra step ratios of uniform layers could be reduced by adding a few fields at key decision points without extending the learning times. Despite its success in improving the extra step ratios, this method did not result in optimal solutions. Furthermore, consistent with experiment 1, adding cells significantly increased learning times only when the resulting distribution had substantially more cells (percentually) than the original distribution.

Experiment 4 proposed that field sizes in optimal distributions should be proportional to the distance to the closest subgoal. Subgoals include the goal itself and places where the robot is forced to change directions, such as near obstacle corners. Results showed that non-uniform multi-scale distributions used fewer cells, learned faster, and reached better final extra step ratios. Notably, although the advantages over the uniform distributions decreased when adding obstacles, distributions were manually designed, and automatic methods will likely find better solutions using fewer cells.

While all experiments were performed with and without eligibility traces, traces did not affect the overall results for experiments 1 and 2. In contrast, when not using traces in experiments 3 and 4, agents using single-scale distributions with field sizes between 20 and 42 cm had trouble learning mazes that required precision, resulting in final trial trajectories between 30 and 60 times longer than the optimal paths. Adding eligibility traces solved this issue, reducing their lengths to at most twice the optimal paths. Therefore, these results suggest that the difficulties in learning the mazes were not the result of limited representational abilities of sparse distributions.

### 5.4. Main takeaways and observations

The main takeaways of our experiments are how the different field sizes along the dorsoventral axis interact with obstacles and how they can be arranged in non-uniform multi-scale distributions to optimize all metrics simultaneously. As an added benefit, non-uniform distributions can potentially increase the number of memories recalled by a robot as fewer cells are required to solve a task. In turn, this may allow the robot to learn more tasks or details.

Although our experiments suggest that the best results are achieved using very little redundancy (overlap between place fields), this is not necessarily the case as we did not use any noise. Omitting noise allowed us to simplify the analysis of the model's theoretical capabilities, but more redundancy could help filter noisy cell activity or prevent memory loss by cell decay in real scenarios.

Although our work did not use hierarchical reinforcement learning (HRL), the proposed space representation shares ideas similar to HRL and could be used to complement such algorithms. HRL speeds up learning by breaking tasks into smaller subtasks (each with a subgoal) that are learned independently along with a method for switching between them (Sutton et al., 1999; Barto and Mahadevan, 2003). Similarly, our model also speeds up learning although by adapting the space representation to the particular environment. As the multi-scale model distributes place cells concentrically around subgoals using higher densities near subgoals, the proposed space representation could naturally lend itself to further speed up HRL algorithms. Additionally, the representation could also be used to enhance automatic subgoal discovery. Finding useful subgoals is a difficult task in HRL (McGovern and Barto, 2001; Goel and Huber, 2003; Botvinick, 2012). With the multi-scale model, subgoal discovery could be performed by finding areas with high concentrations of smaller fields. In our algorithm, place fields concentrate around obstacle corners as we assumed obstacle corners to be subgoals (which conforms with rat experiments Shamash and Branco, 2021; Shamash et al., 2021). Nonetheless, an HRL algorithm may want to use only a subset of these locations. Thus, the suggested space representation may hint useful subgoals, but another mechanism could further filter them out.

### 5.5. Biological context

Our model was inspired by differences in the dorsoventral axis of the hippocampus. In building the model, as several other models, we used reinforcement learning to simulate the brain's learning process based on observations where dopaminergic neurons predict error signals as temporal differences (James et al., 1994; Schultz et al., 1997; Doya, 2008). Additionally, we assumed that place cells provide the state in a locale learning system and that both the dorsal and ventral hippocampus are involved in spatial navigation (de Hoz et al., 2003; Harland et al., 2017; Contreras et al., 2018).

Based on experimentation, our model predicts the possible effects of inactivating dorsal or ventral place cells. Inactivating ventral place cells should increase learning times and reduce the ability to generalize actions. Furthermore, it may also reduce the number of tasks or the amount of detail that an animal can learn as inactivating ventral cells will require substantially more dorsal cells to encode a task. On the other hand, inactivating dorsal place cells should increase learning time in obstacle-rich environments as the representation will rely on larger fields. Either way, our experiments showed that any scale could be used to learn a task, provided enough cells are used. Thus, deactivating either dorsal or ventral place cells should not prevent an animal from learning, but it should affect how they react to different obstacle numbers as they rely more heavily on one representation. Note how obstacles had a more significant effect on larger fields than smaller fields in experiment 1. In Llofriu et al. (2019), the authors analyzed how dorsal or ventral hippocampus deactivation affected the time to complete a spatial navigation task using a computational model in cluttered environments. Similar to our predictions, all agents were able to learn the task, although deactivating either region resulted in longer completion times.

Our model also predicts that place field representations should be denser around subgoals and sparser when further away. Similarly, smaller fields should concentrate around subgoals, and field sizes should increase when further away.

In the available literature, several rat electrophysiological studies have observed varied spatial distribution of place cell fields according to the environment, e.g., higher place cell field concentrations near goals in the dorsal hippocampus (Hollup et al., 2001; Fyhn et al., 2002; Hok et al., 2007; Dupret et al., 2010; Tryon et al., 2017). Additionally, dorsal and ventral hippocampus experiments found that smaller fields aggregate around walls while fewer larger fields are more prevalent in the middle of the maze, with both types seen throughout the complete environment (Harland et al., 2021; Tanni et al., 2022). Importantly, these experiments were performed in mazes without obstacles and fixed goals and, therefore, cannot assess field distribution in relation to them. Although our non-uniform distributions did not have small fields near walls or throughout the maze, the distributions were manually generated to assess the benefits of distributing field sizes based on goals and subgoals, ignoring other factors that might be used to instantiate fields, such as the specific location of visual cues or landmarks. In Harland et al. (2021), there is an extensive number of visual cues, including distal on the room walls and proximal on the maze walls and on the floor itself. This cue-richness may explain the activation of small place fields across the entire environment, including the observation by Harland et al. (2021) of small place fields in the center of the maze, likely because of the floor cues.

### 5.6. Alternative models

We discuss in this section other models that have been used to assess how the different place field sizes might be used for navigation and to make predictions about the spatial distribution of place fields.

In the boundary vector cell model (Burgess et al., 2000; Barry et al., 2006), place cell firing is the result of combining the output of multiple boundary vector cells, which are neurons that activate when a boundary is detected at a given distance and allocentric direction from the rat. This model predicts that smaller place fields should be more numerous than larger place fields and that the concentration of each type should increase when close and far from boundaries, respectively. On the other hand, this model does not explain how place cells are affected by goals nor how they are used for learning. Interestingly, if the boundary vector cell model also incorporated object vector cells (Hoydal et al., 2018) as input for place cells, the resulting distributions might resemble the non-uniform distributions proposed in this paper.

Although our article used place cells to represent the state (current position) in reinforcement learning algorithms, the successor representation model assumes that place cells encode “a predictive representation of future states given the current state” (Stachenfeld et al., 2017). Under this theory, the dorsoventral multi-scale representation is the result of encoding the successor representation using multiple discount factors, which enables using different temporal abstraction levels for decision-making.

### 5.7. Future work

As part of future work, we plan to evaluate the model with autonomous robots in physical environments to assess the effects of noise in optimal distributions. In order to achieve this goal, some optimizations would be required. First, we need to activate place cells driven by sensory-motor cues rather than global positioning. Second, place field representations should not be manually generated. Instead, place fields should automatically adapt to the environment according to the distance to subgoals. This could be done either by generating a single multi-scale layer for each specific environment (as in this paper) or by generating multiple single-scale layers, each covering the entire maze (such as in the uniform multi-scale distributions) and then choosing which layers to activate based on environmental cues. In either case, our work suggests that place fields should get smaller near the goal, but it may be argued that its location is a priori unknown. While this may be true during the initial trials, the position of the goal should be known later on, as suggested by electrophysiological studies that found neurons that encode the distance and egocentric angle to the goal even when not seen (Deshmukh and Knierim, 2013).

Our current model assigns a single place field to each place cell, but recent experiments in large environments show this is not the case (Fenton et al., 2008; Rich et al., 2014; Lee et al., 2020; Eliav et al., 2021; Harland et al., 2021). Instead, experiments show that both dorsal and ventral place cells can have multiple fields of different sizes, forming a multi-field multi-scale space representation. Additional future work should update the model to reflect the corresponding findings.

## Data availability statement

The original contributions presented in the study are included in the article/Supplementary material, further inquiries can be directed to the corresponding author.

## Author contributions

PS developed the model, carried out the simulations, and wrote the manuscript with input from all authors. J-MF and AW conceived the study and were in charge of the overall direction and planning. All authors contributed to the article and approved the submitted version.

## Footnotes

^1^SCS-https://github.com/biorobaw/scs.

^2^Multiscale project-https://github.com/biorobaw/Multiscale-V2.

^3^CIRCE-https://wiki.rc.usf.edu/index.php/CIRCE_Hardware.
