# Supplementary material for: Adapting hippocampus multi-scale place field distributions in cluttered environments optimizes spatial navigation and learning
Source: Front Comput Neurosci. 2022 Dec 12;16:1039822. doi: 10.3389/fncom.2022.1039822 (PMC9792172; doi:10.3389/fncom.2022.1039822)
Supplement: Supplementary file 1 [file Data_Sheet_1.pdf]

Supplementary Material

1 MAZES

In total, we used 63 mazes of identical dimensions (2.2 m by 3 m) for the experiments, each with its own goal, starting locations, and obstacles. Of the 63 mazes, 3 were manually set up, and 60 were generated automatically.

Handmade mazes included mazes 0, 1, and 2 with 0, 2, and 6 differently sized obstacles, respectively. These mazes, illustrated in figure 1b, were mainly used to assess non-uniform place cell distributions. Note that mazes 0 and 1 differ only by the presence of 2 additional obstacles forming a small gap. This design was chosen to assess possible issues arising from using large place cells to generalize information from one side of the maze to the other. Additionally, maze 1 was also designed to force multiple paths to go through the same 'subgoal' (the gap).

The other 60 mazes were generated automatically by sampling 10 random configurations of 10, 20, . . . , and 60 25 cm long obstacles and were mainly used to assess how different obstacle configurations affect place cell distributions. Accordingly, maze names were formatted so that the first two digits indicate the number of obstacles, and the last digit indicates the configuration. For example, maze 108 is the 8th configuration including 10 obstacles. All automatically generated mazes shared the same goal and starting locations, but obstacles were generated independently by incrementally sampling obstacle centers and orientations from uniform distributions. Obstacles were required to be at least 10 cm away from each other, and their center had to be 12.5 cm away from the walls to prevent intersections and allow the agent (simulated rats) to move between obstacles. Thus, resampling was done if necessary until these criteria were met. Sample autogenerated mazes are shown in figure 1b.

Using multiple starting locations per maze increases the difficulty of learning a policy that optimizes all paths simultaneously by requiring the model to learn different actions in nearby regions of space. This idea is exemplified in appendix figure S1, which shows two identical empty mazes with the same goal but different starting locations. In the image, the maze with a single starting location can use a single place cell to learn the task, while the maze with 2 starting locations requires at least 2 place cells. Additionally, requiring a second place cell increases the overlap between cells. Thus, the policy learned by one place cell becomes correlated with the policy learned by the other. As a result, this also increases the algorithm's complexity as a change in one cell destabilizes the policy learned by the other, making the algorithm more unstable, as explained by (Kretchmar and Anderson, 1997).

2 EXPERIMENT 1 RESULTS

2.1 Uniform single-scale distributions

Appendix table S1 shows all distributions used in the experiment. As observed in the table, not all uniform grids were used with every scale, as some combinations generate representations that do not cover the entire maze. Thus, we only combined field sizes with grids that had equal or more cells than the MCDs.

To illustrate the redundancy of each place cell distribution, or alternatively, of its overlap between fields, appendix figure S4 plots the average number of cells that are active at any point in the maze. To measure the redundancy of a layer, we tessellated the maze into 1 mm squares, counted the number of fields intersecting

each square, and averaged the results. Importantly, increasing either the number of cells or the field sizes of a layer increases its redundancy. Also, although hard to observe in the image, the minimum redundancy of each scale is close to 1 and is achieved by the respective MCD distributions as they use the least number of cells to cover the maze. A redundancy of 1 means that each position is represented on average by a single place cell.

2.2 Exploration times

In experiment 1, observing that adding obstacles generally led to longer learning times, we considered whether this could be explained by longer exploration times during initial trials. To assess this possibility, in appendix figure S7, we plotted the extra step ratios during the first trial for each number of obstacles. Doing a Kruskal Wallis test (Ostertagová et al., 2014) followed by a Dunn test with Bonferroni corrections (Armstrong, 2014; Dunn, 1964) showed no significant difference ($p > 0.07$) between groups with 0, 10, 20, and 40 obstacles and no significant difference ($p > 0.07$) between groups 30, 50, and 60. Thus, it seems unlikely that the increase in learning time could be explained by longer initial exploration times.

2.3 Optimal number of cells

In terms of learning times, the optimal number of cells for each scale is given by the distribution using the least number of cells. This was observed in section 4.1.3, where the learning times for a given scale decreased with the number of cells. Exceptions included distributions with very few cells that had issues learning the mazes.

On the other hand, in section 4.1.4, we observed that the optimal number of cells in terms of extra step ratios varied according to the scale and the number of obstacles; thus, we plotted this information in appendix figure S10. Appendix figure S10 shows two plots. The first plot shows the optimal number of cells (defined as the number of cells with the lowest mean extra steps ratio) for each scale for each number of obstacles. The second plot shows the same information but averages all scales to assess the overall trend as the maze complexity increases.

As observed in figure S10, results show that most scales reach their optimal value between 140 and 315 cells, with larger scales requiring fewer cells than smaller scales. Furthermore, as the number of obstacles increases, the optimal number of cells of each scale tends either to increase or to remain constant. This is further evidenced by the right plot in figure S10, where we observe that the average optimal number of cells increases with the number of obstacles.

3 EXPERIMENT 3 RESULTS - QUALITATIVE DIFFERENCES

To qualitatively assess the effects of adding cells around the goal and the gap, in appendix figure S12, we plotted the paths during the final trial performed by a group of agents before and after adding the cells. Focusing on the areas circled in red, the image shows that adding cells around the goal results in trajectories that move straighter towards the goal avoiding trajectories such as first moving east and then north or overbearing north and then correcting. Focusing on the areas circled in yellow, we make two observations. First, adding cells around the gap allowed the models to bypass the gap when they were previously getting stuck. Second, before adding the cells, the paths from different starting locations would first be merged before moving across the gap. After adding the cells, the model separated the trajectories allowing them to learn more efficient paths. This shows how adding place cells in one region may influence the decision-making process in areas far away from the fields of the cells. This also shows that the paths learned by the model are susceptible to the placement of the place cells.

ALGORITHMS

Algorithm 1 Multi-scale model RL

```
Get position and reward (x⃗_t, r_t)
Compute place cell activity (P_it) - section 3.2
if t > 0 then
    Compute bootstrap value (V'_t) - section 3.6
    Compute RL Error (δ_t) - section 3.6
    Update weights (V_it, Q_ij) - section 3.6
end if
Compute the state value (V_t) - section 3.3
Compute action preferences (Q_jt) - section 3.3
Choose next action (a_t) - section 3.4
Update traces (z_it, z_ijt) - section 3.5
Perform action
t ← t + 1
```

TABLES

Scale	MCD	35 (5x7)	140 (10x14)	315 (15x21)	560 (20x28)	875 (25x35)	1230 (30x41)	168 (35x48)	2200 (40x55)
4	2200 (40x55)								✓
8	609 (21x29)					✓	✓	✓	✓
12	280 (14x20)			✓	✓	✓	✓	✓	✓
16	165 (11x15)			✓	✓	✓	✓	✓	✓
20	117 (9x13)		✓	✓	✓	✓	✓	✓	✓
24	70 (7x10)		✓	✓	✓	✓	✓	✓	✓
28	54 (6x8)		✓	✓	✓	✓	✓	✓	✓
32	54 (6x8)		✓	✓	✓	✓	✓	✓	✓
36	54 (6x8)		✓	✓	✓	✓	✓	✓	✓
40	35 (5x7)	✓	✓	✓	✓	✓	✓	✓	✓
44	35 (5x7)	✓	✓	✓	✓	✓	✓	✓	✓
48	24 (4x6)	✓	✓	✓	✓	✓	✓	✓	✓
52	24 (4x6)	✓	✓	✓	✓	✓	✓	✓	✓
56	24 (4x6)	✓	✓	✓	✓	✓	✓	✓	✓

Table S1. Uniform distributions. The table shows all the uniform distributions used in the first experiment. Column “Scale” indicates the place field radii in centimeters. Column “MCD” (minimum coverage distribution) indicates the shapes of the uniform distributions that use the least number of cells to cover the maze with the respective scales. The shapes are denoted by the number of cells, followed by the number of columns and rows in parenthesis. All MCDs were included in the experiment. The remaining columns indicate other distribution shapes denoted in the same format. Tickmarks indicate distributions used in the experiment resulting from combining the given row (scale) and column (shape). Note that missing tickmarks correspond with distributions that cannot cover the maze as they use fewer cells than the MCD. Also, note that the MCD for scales 4, 40, and 44 coincide with one of the other distributions. Thus, 97 different uniform distributions were used in total (one for each tick in the table, plus one MCD for each scale, minus 3 repeated distributions).

FIGURES

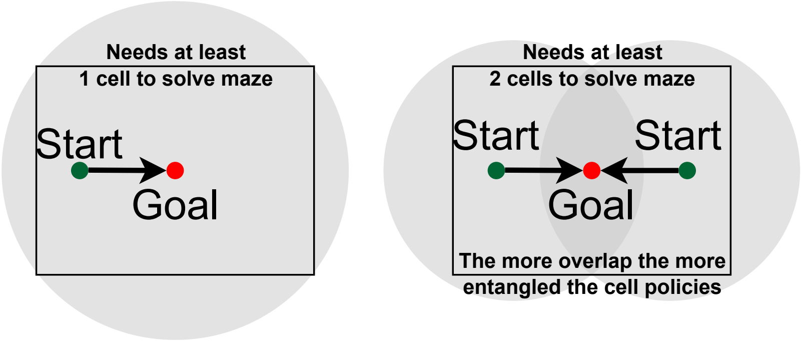

Figure S1. Task complexity. The figure illustrates two identical mazes with the same goal but different starting locations. The left maze has a single starting location, and thus it can be solved using at least one cell that learns to go right. The right maze adds a second starting location on the opposite side of the maze. The model now requires at least 2 cells to learn different actions on each side, increasing the complexity of the model. Additionally, having overlapping fields means that the policies learned by each cell become entangled as a change in the policy learned by one field results in a change in the policy learned by the other. This makes the algorithm more unstable and further increases the difficulty of learning the task.

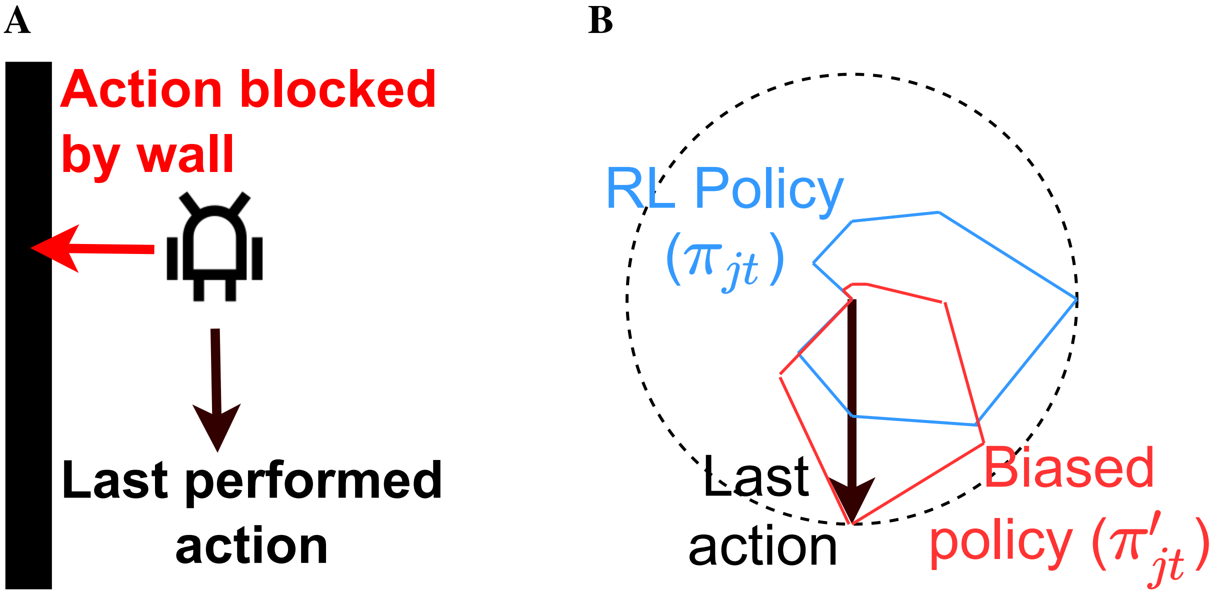

Figure S2. Blocked actions and motion bias. **(A)** Illustration of a scenario after the robot performed a downward motion (black arrow). The robot cannot move left due to the presence of a wall (red arrow). **(B)** Sample polar plots illustrating the actor's policy (probability of performing each action) before (blue plot) and after (red plot) adding the motion bias in scenario (A). In both plots, the probability of moving left is 0 as the action is blocked by the wall. After adding the motion bias (red plot), the action probabilities are increased for downward motions and decreased for upward motions as the bias increases the likelihood of performing actions similar to the last action (black arrow in the example). The motion bias avoids switching directions too often by making the model more likely to keep choosing similar actions.

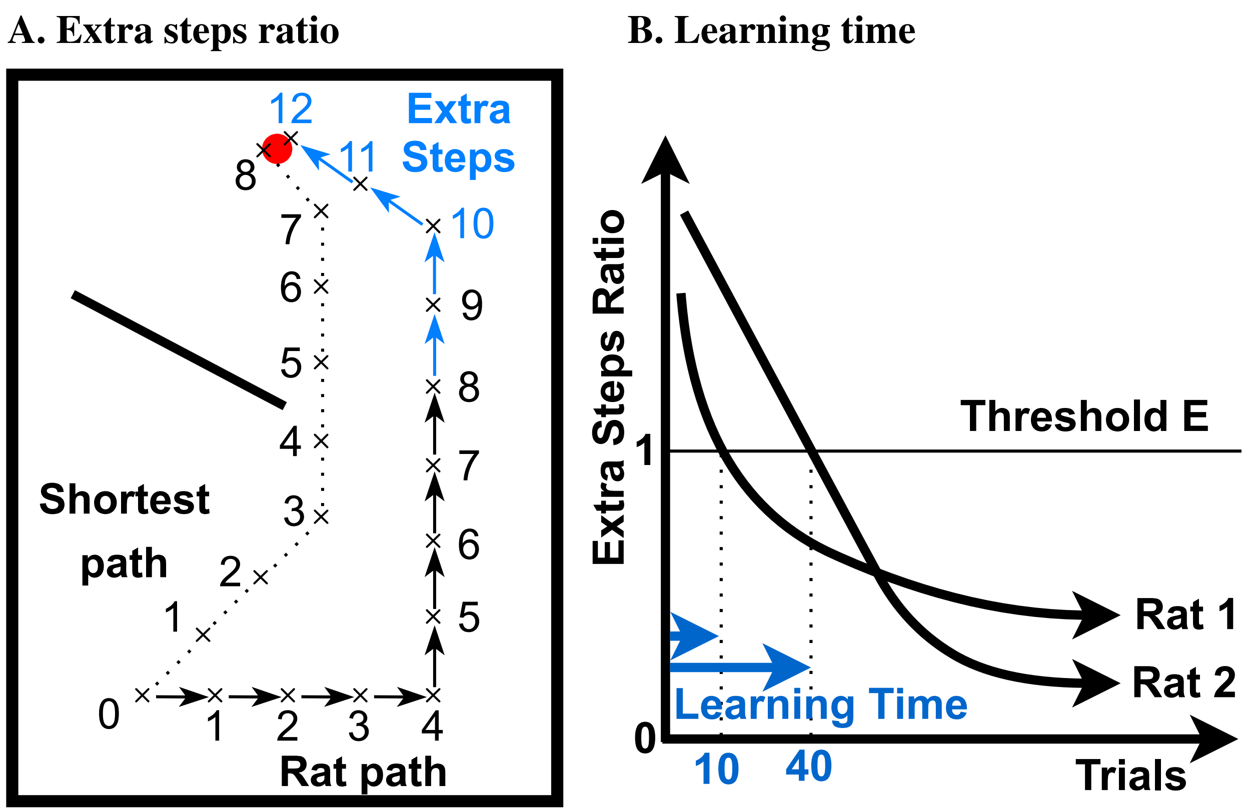

Figure S3. Metric illustrations. **(A)** Extra steps ratio. The figure shows the path followed by an agent (arrows) and the corresponding shortest path (dotted lines). The agent performed 4 extra steps (blue arrows) as it took 12 steps to reach the goal, but only 8 were required. To calculate the extra steps ratio, we normalize results by the shortest path, leading to an extra steps ratio of 0.5 ($\frac{12-8}{8}$). **(B)** Learning time. The figure shows an illustration of the extra steps ratio per trial for two simulated agents. The learning times are defined as the number of trials to reach the predefined performance level (the threshold). Accordingly, the learning times for agents 1 and 2 are 10 and 40 trials.

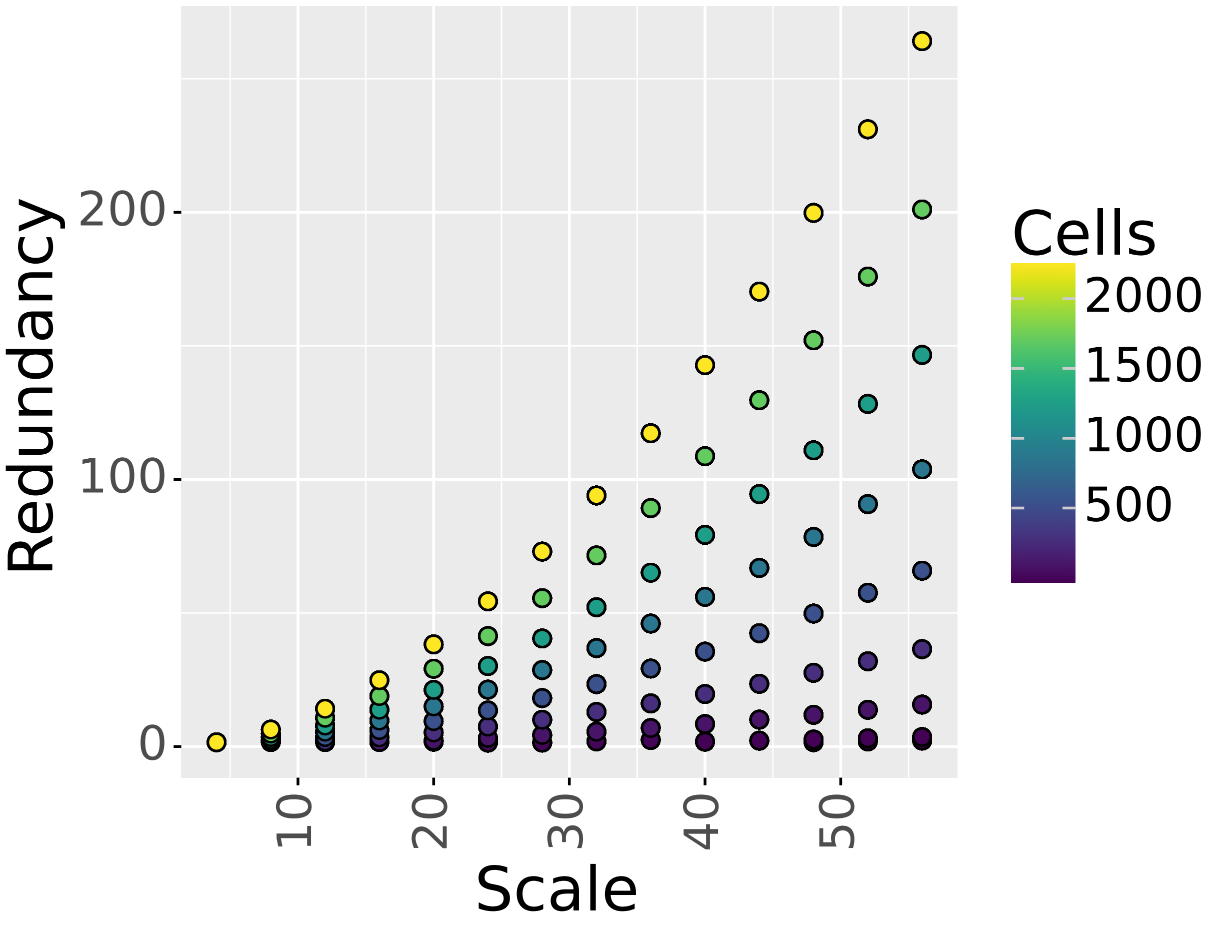

Figure S4. Redundancy of uniform layers. The plot above shows the average number of cells (y-axis) that are active at any point on the maze for each uniform layer of place cells. Uniform layers are determined by the field size (x-axis) and the number of place cells (color). Note that the redundancy increases both with the number of cells and the field sizes. Additionally, the minimum redundancy is close to 1 for all scales and is achieved when using the least number of cells to cover the maze.

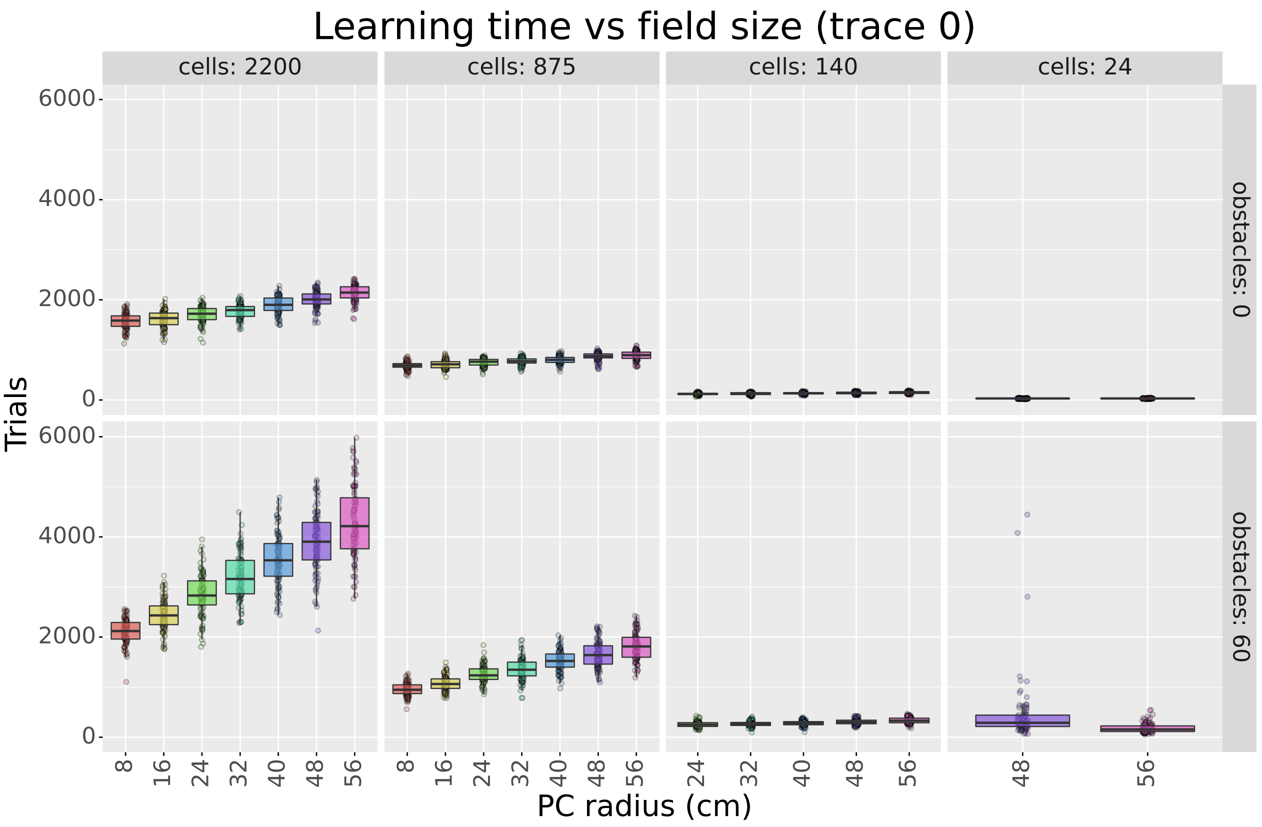

Figure S5. Learning time as a function of the field size in the first experiment. The figure compares the learning time box plots of seven field sizes for different cell numbers (columns) and obstacle numbers (rows). For 24 and 140 cells, some field sizes are missing as the resulting layers would not cover the entire maze.

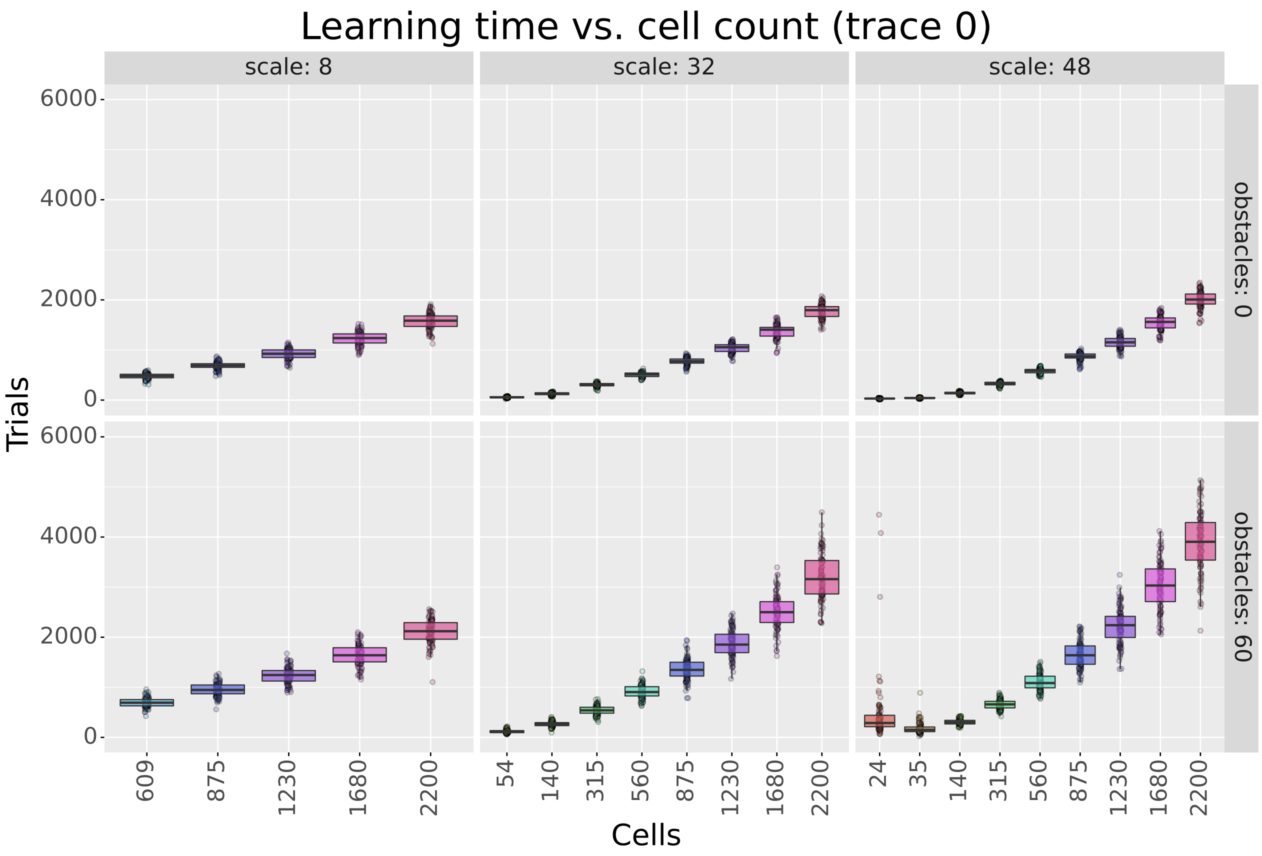

Figure S6. Learning time as a function of the number of cells in the first experiment. The figure compares the learning time box plots of nine numbers of cells for different field sizes (columns) and obstacle numbers (rows). For scales 8 and 32, some cell numbers are missing as the resulting layers would not cover the entire maze.

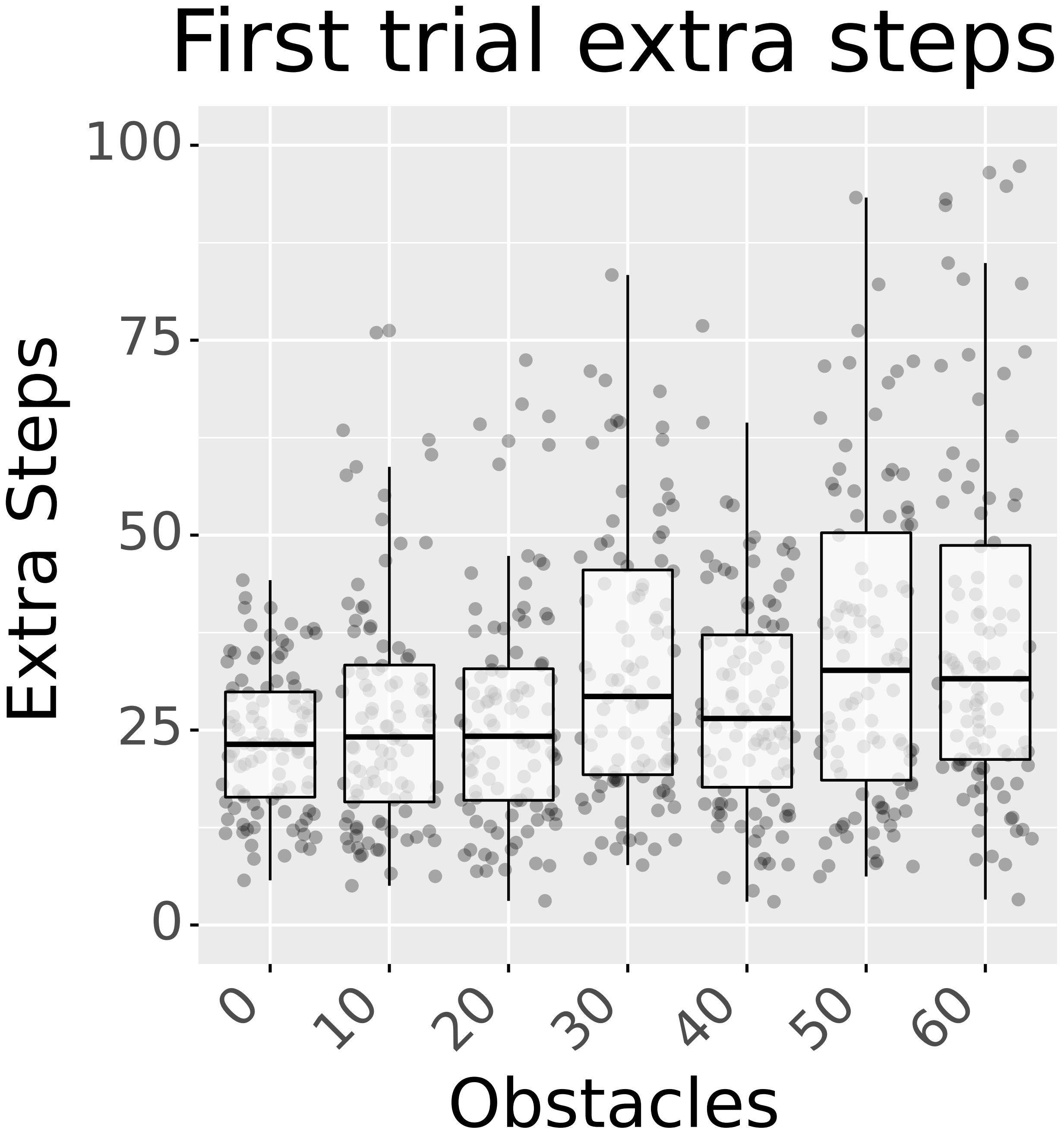

Figure S7. Extra step ratios of the first trial for the different number of obstacles in experiment 1.

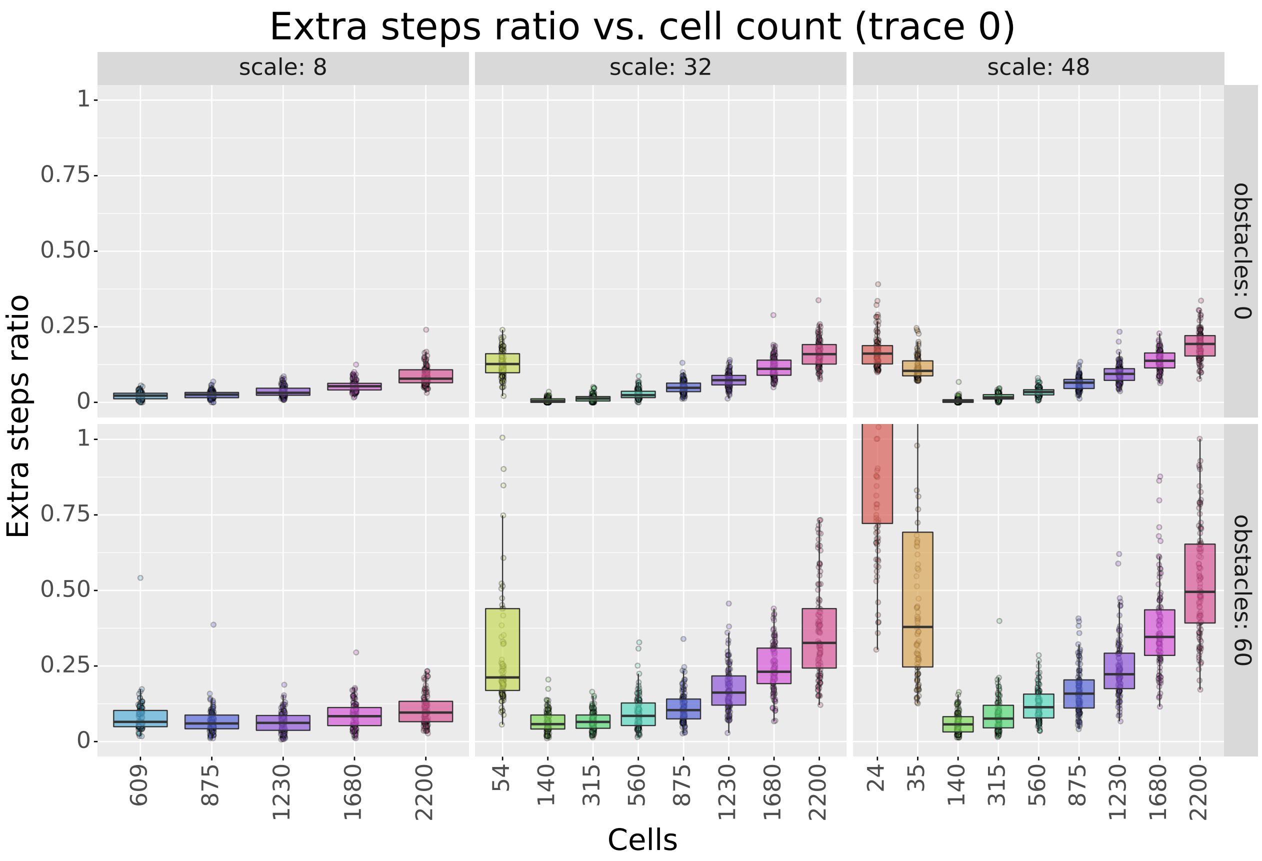

Figure S8. Extra step ratios as a function of the cell number in the first experiment. The figure compares the extra steps ratio box plots of nine cell numbers for differentfield sizes (columns) and obstacle numbers (rows). For scales 8 and 32, some cell numbers are missing as the resulting layers would not cover the entire maze.

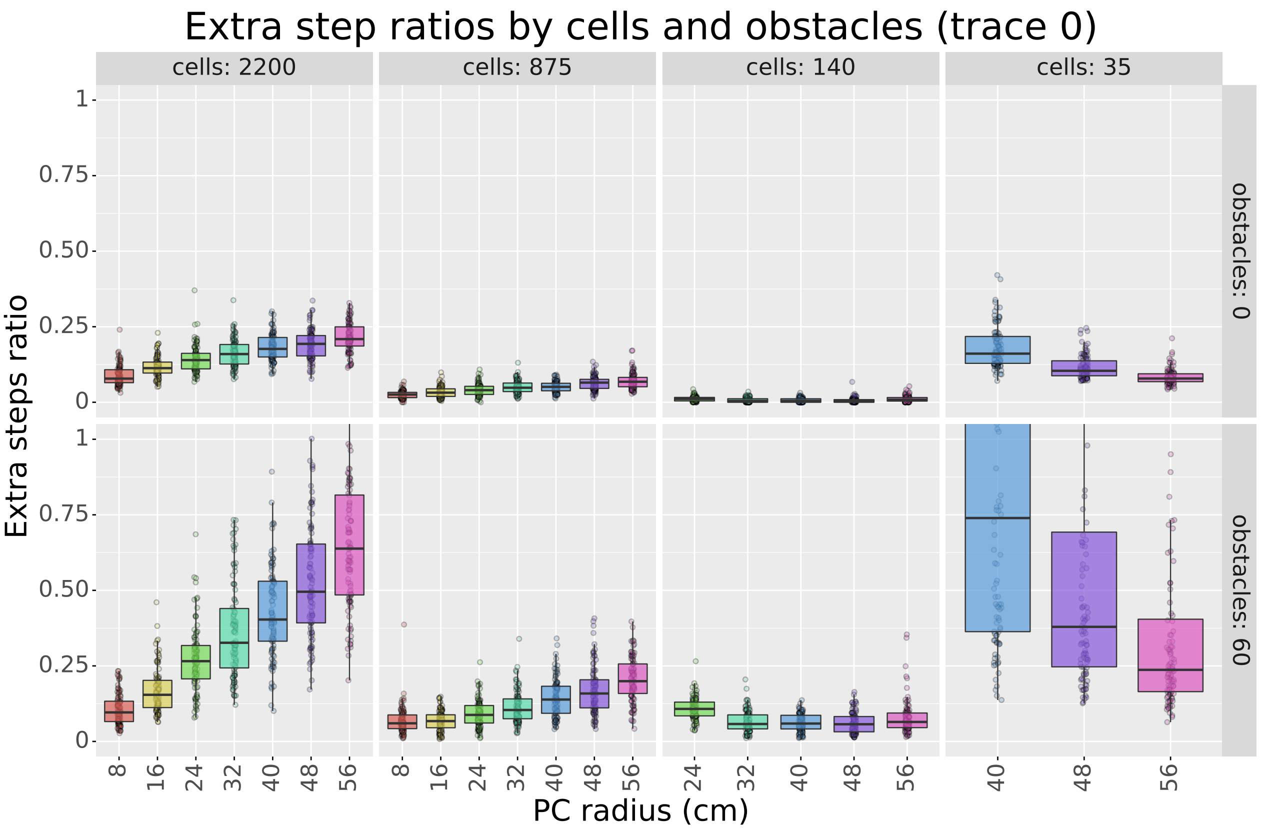

Figure S9. Extra step ratios as a function of the field size in the first experiment. The figure compares the extra steps ratio box plots of seven field sizes for different cell numbers (columns) and obstacle numbers (rows). For 35 and 140 cells, some field sizes are missing as the resulting layers would not cover the entire maze.

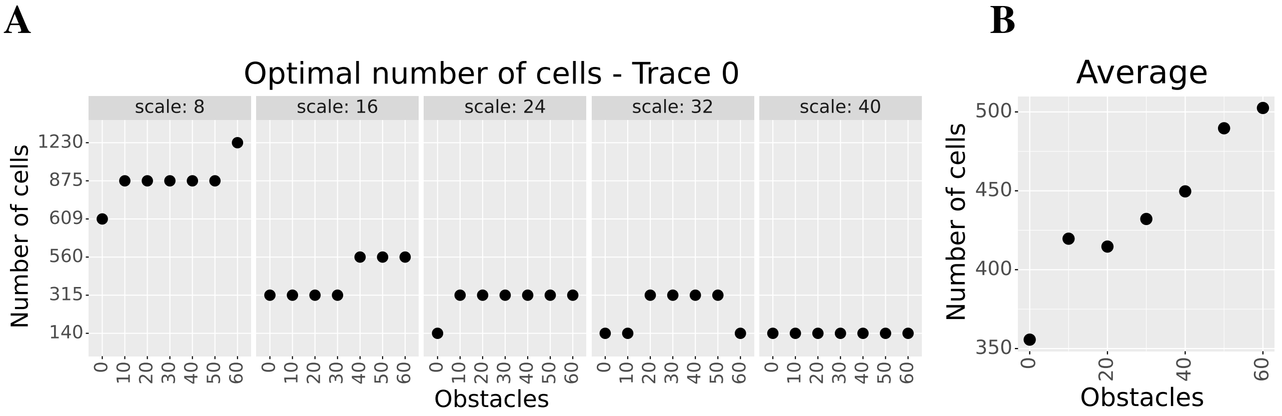

Figure S10. Optimal number of cells for reducing mean extra steps. **(A)** Optimal number of cells vs. number of obstacles for five sample scales. **(B)** General trend after averaging results from all scales (including scales not shown). In general, more obstacles required more cells to achieve optimal results. Also, panel a shows that larger scales required fewer cells to achieve their peak result.

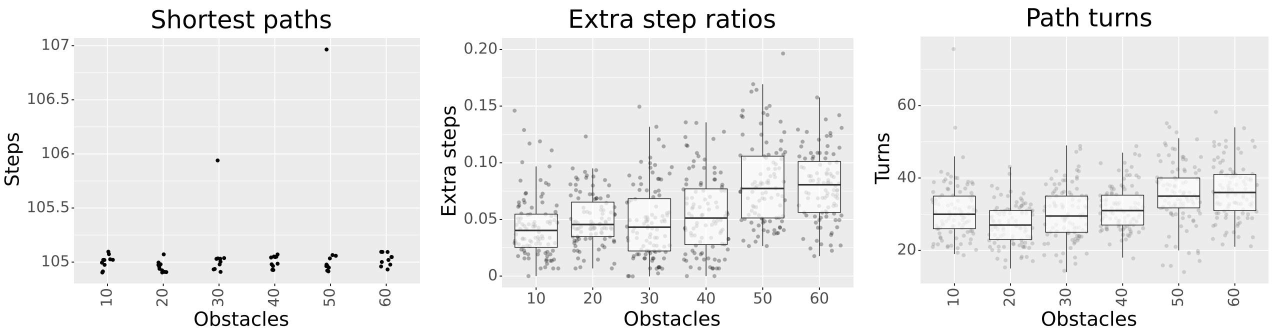

Figure S11. Final trial paths data. The plots above show information about the optimal paths and the paths performed by the agents during the last trial of the uniform multi-scale experiment. The left plot shows the lengths of the optimal paths, the middle plot shows the extra step ratios during the final trial, and the right plot shows the number of times that the agents performed a turn (change in the direction of motion). Note that in the first plot, although results should be integers, we added a small displacement to each data point in order to see overlapping data points.

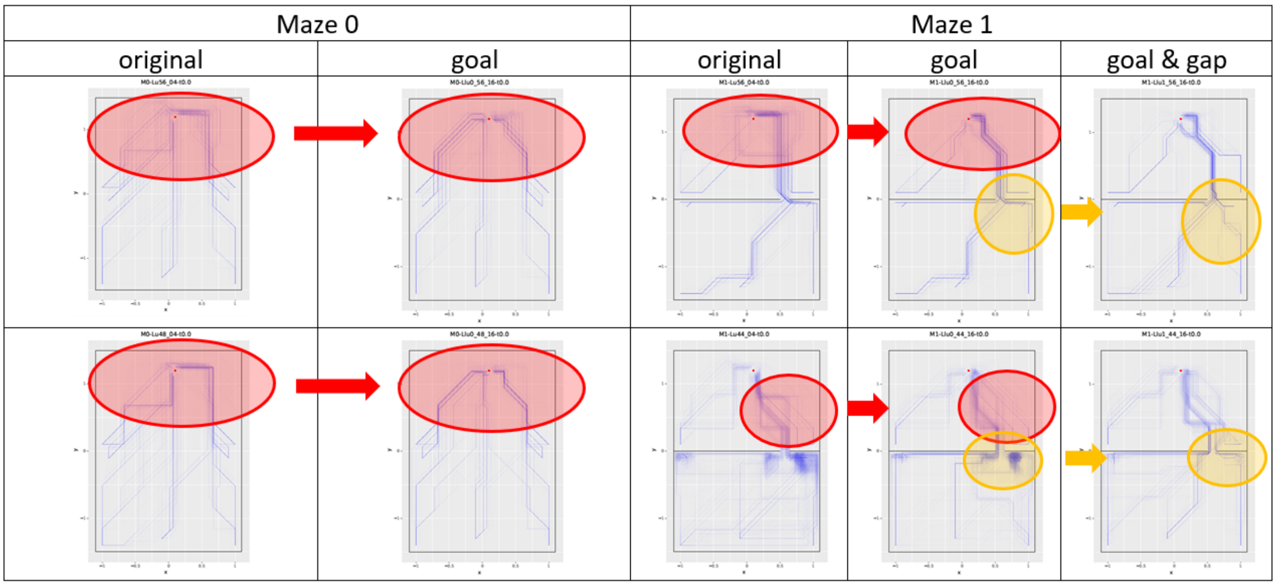

Figure S12. Rat paths before and after adding cells at the goal and the gap in mazes 0 and 1. Each plot shows the superposition of the paths performed during the final trial of the 100 agents corresponding to the respective group. The upper row corresponds to scale 56 for both mazes, while the lower row corresponds to scale 48 in maze 0 and scale 44 in maze 1. Circled areas indicate areas to focus on while comparing the changes. Red areas indicate changes related to the goal, while yellow areas indicate changes related to the gap.

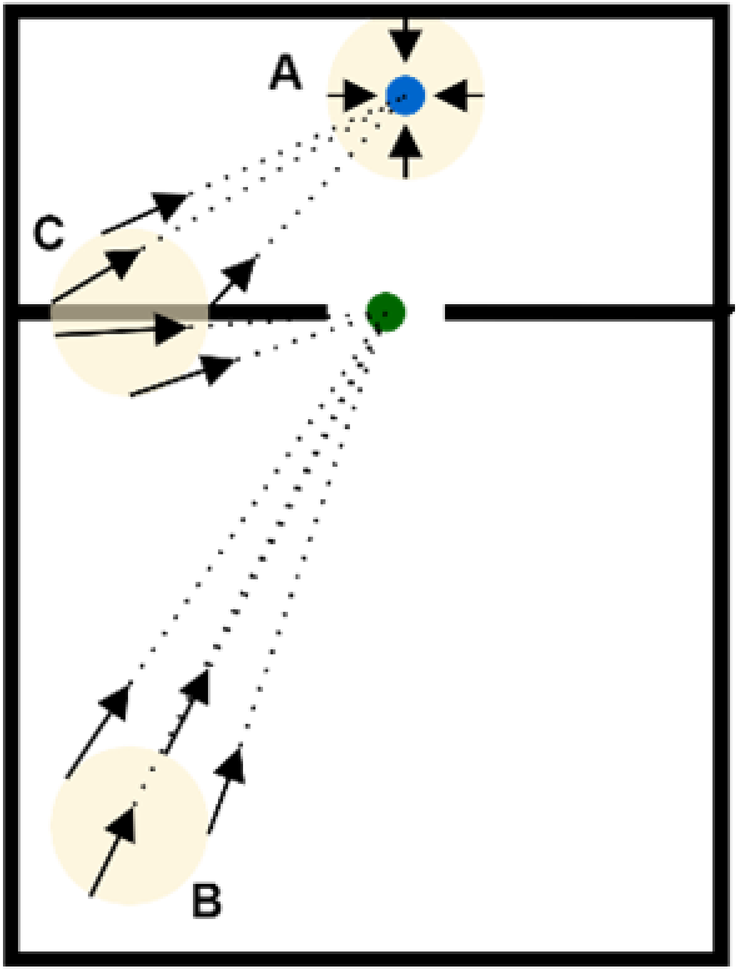

Figure S13. Policy change vs. distance to subgoal. The figure represents the optimal action policy to reach the goal (blue dot) from multiple points in the three shaded areas shown in the figure. The green dot represents a subgoal that must be reached if starting from the bottom half of the maze. Comparing shaded regions A and B, we observe that the further away we are from the next subgoal, the more similar the actions are within the same area (all arrows in B point nearly in the same direction, while all arrows in A point in different directions). This suggests that fewer larger cells could be used to encode area B as compared to A. Also, note that a wall intersects area C generating a discontinuity between both sides of the wall. Although this may suggest that we want either smaller or denser fields around C, this is only true if place fields activate across the wall.

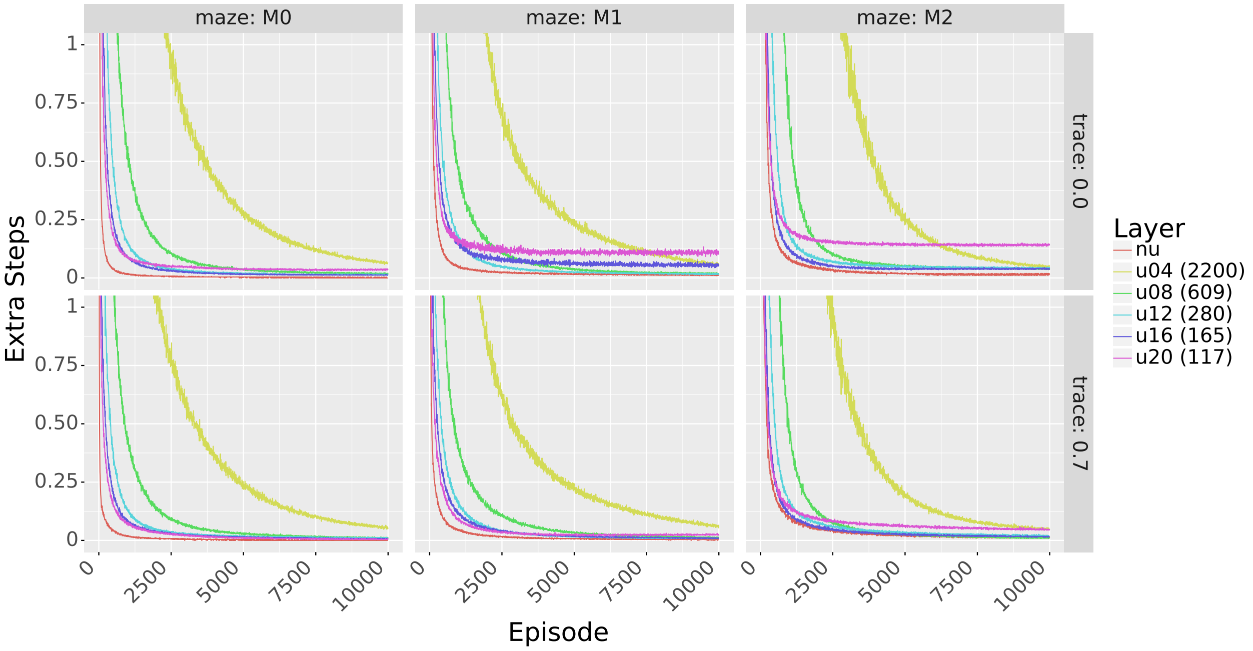

Figure S14. Extra step ratios vs. trial. The plots above compare the 'extra steps ratio vs. trial' time series for uniform and non-uniform distributions. Rows show results using different traces, while columns show the results in each maze. Layer prefixes 'u' and 'nu' indicate uniform and non-uniform distributions. For uniform distributions, the two digits following the prefix indicate the scale, while the number inside parentheses indicates the number of cells. Non-uniform distributions had 38 cells in maze 0, 79 in maze 1, and 117 in maze 2.

REFERENCES

Armstrong, R. A. (2014). When to use the Bonferroni correction. *Ophthalmic and Physiological Optics* 34, 502–508. doi:https://doi.org/10.1111/opo.12131

Dunn, O. J. (1964). Multiple comparisons using rank sums. *Technometrics* 6, 241–252. doi:https://doi.org/10.1080/00401706.1964.10490181

Kretchmar, R. M. and Anderson, C. W. (1997). Comparison of CMACs and radial basis functions for local function approximators in reinforcement learning. *IEEE International Conference on Neural Networks - Conference Proceedings* 2, 834–837. doi:10.1109/ICNN.1997.616132

Ostertagová, E., Ostertag, O., and Kováč, J. (2014). Methodology and application of the Kruskal-Wallis test. *Applied Mechanics and Materials* 611, 115–120. doi:10.4028/www.scientific.net/AMM.611.115
